# Converging forms: an examination of sub-Arctic, circumarctic, and Central Asian *Ranunculus auricomus* agg. populations

**DOI:** 10.3389/fpls.2024.1415059

**Published:** 2024-06-17

**Authors:** John Paul Bradican, Salvatore Tomasello, Judith Vollmer, Elvira Hörandl

**Affiliations:** ^1^ Department of Systematics, Biodiversity and Evolution of Plants (with herbarium), Albrecht-von-Haller Institute for Plant Sciences, University of Göttingen, Göttingen, Germany; ^2^ Georg-August University School of Sciences (GAUSS), University of Göttingen, Göttingen, Germany; ^3^ Department of Physics, Chemistry and Biology, Linköping University, Linköping, Sweden

**Keywords:** apomixis, polyploidy, hybridization, geographical parthenogenesis, cold adaptation, *Ranunculus*

## Abstract

**Introduction:**

Phenotypic complexity in species complexes and recently radiated lineages has resulted in a diversity of forms that have historically been classified into separate taxa. Increasingly, with the proliferation of high-throughput sequencing methods, additional layers of complexity have been recognized, such as frequent hybridization and reticulation, which may call into question the previous morphological groupings of closely related organisms.

**Methods:**

We investigated Northern European, Asian, and Beringian populations of *Ranunculus auricomus* agg. with phylogenomic analysis of 736 genes and 27,586 SNPs in order to deduce the interrelatedness and hybrid origin of this phenotypically and taxonomically complicated group from Europe characterized by a history of hybridization, polyploidy, apomixis, and recent radiation. The ploidy levels and the reproductive mode of the Northern European populations were assessed via flow cytometric seed screening. In addition, in order to examine the phenotypic plasticity of the dwarf forms previously described as species and summarized as the *Ranunculus monophyllus* group, we conducted climate chamber experiments under cold (northern) and warm (temperate) conditions.

**Results:**

The Northern European populations are tetra- to hexaploid and propagate primarily through apomixis. The complex is characterized by highly reticulate relationships. Genetic differentiation of the main clusters has occurred between the above-mentioned geographical regions. We find evidence for the hybrid origin of the taxa in these areas with differing genomic contributions from the geographically nearest European sexual progenitor species. Furthermore, polyphyly in the taxa of the *R. monophyllus* group is supported. Experiments show low lability in the traits associated with the *R. monophyllus* group.

**Discussion:**

We conclude that multiple adaptations of hybrids to colder climates and shorter vegetation periods have shaped the phenotypes of the *R. monophyllus* group, and we suggest a formal classification as nothotaxa within the *R. auricomus* group.

## 1 Introduction

The broad- and fine-scale gradations in the morphological diversity present within the angiosperms has provided the basis for the study of the systematics of this branch of life (Endress et al., 2000; Rouhan and Gaudeul, 2014). This history of observation has provided key and long-lasting demarcations between the types and forms of plants, accompanying cultural, medicinal, agricultural, and scientific development (Hill, 1915; Li, 1974; Soltis et al., 2019). In recent decades, the development of DNA sequencing and the progress in sequence interpretation methods have continued to refine our understanding of the tree of life, resulting in changes to some long-standing concepts of phylogenetic relationships (The Angiosperm Phylogeny Group, 2003; Saarela et al., 2007). This general trend extends to the intraspecific level, where high-throughput sequencing techniques have generated large quantities of data, contributing to the recent advancements in the understanding of cryptic diversity and the often intricate interrelationships present in species complexes (Li et al., 2020; Boucher et al., 2021; Bobrov et al., 2022; Buck and Flores-Rentería, 2022). Both species complexes and species with high phenotypic plasticity and diversity present challenges to the clear demarcation between groups, particularly when accompanied by a history of hybridization and whole-genome duplication (Hörandl, 2022). Such lineages are also often associated with recent radiations, with varying degrees of range expansion (Liu et al., 2017; Karbstein et al., 2021; Romeiro-Brito et al., 2023). In the case of groups with a large distribution range and a broad range of morphological variation, it can be considered whether these morphological differences represent an increased adaptation to the broader range of local conditions (Hoffmann et al., 2010; Pease et al., 2016).

Physiological change as an adaptation to novel environments may present itself as a novel phenotype, but this may also be a result of the aforementioned hybridization and/or polyploidy (Ramsey, 2011; Shimizu-Inatsugi et al., 2017; Van De Peer et al., 2021). Such changes often occur in the context of the expansion of a lineage into newly available habitats, whether the result of large-scale landscape-altering processes such as climate change or long-distance dispersal to islands, among other possibilities (Kapralov et al., 2013; Schwarzer and Joshi, 2019). These events may be particularly climactic, facilitating rapid expansions of formerly more restricted lineages (Karbstein et al., 2021). Rapid radiations have been a subject of interest for researchers, owing to the pace of diversification sometimes associated with these events (Schenk, 2021). Aside from the natural and artificial processes that open the door to rapid expansion, other characteristics may facilitate an organism’s ability to cover ground quickly, for example reproductive characteristics such as selfing, vegetative propagation, and apomixis and the development of seeds in lieu of meiotic division and recombination (Bierzychudek, 1985; Asker and Jerling, 1992; Kearney, 2005; Kirchheimer et al., 2018). This combination of asexual propagation and rapid expansion in an organism’s range has been dubbed geographic parthenogenesis (Vandel, 1928; Hörandl, 2006; Tilquin and Kokko, 2016).

Evidence for recent radiations can be found in many of the plant lineages resident to the Arctic biome (Guggisberg et al., 2009; Hoffmann et al., 2010; Hörandl, 2023). Diversification of the lineages of *Artemisia* and *Ranunculus* occurred in this region, contributing to the rich flora of at least 2,300 species (Hoffmann et al., 2010). In addition, a high incidence of polyploidy has been observed in the Arctic flora, increasing with latitude (Brochmann et al., 2004). This can be partly attributed to the climatic oscillations that occurred during the Quaternary, driving allopatric speciation during glacial accretion, and the development of contact zones as glaciers ebbed, leading to hybridization and whole-genome duplication events (Kadereit and Abbott, 2021). It has been debated whether certain vigor-inducing effects observed in hybrid plants are a factor for their high frequency in the Arctic flora, such as an increase in heterozygosity in selfing or clonal populations (Brochmann et al., 2004). Moreover, for some lineages, expansion into and across the sub-Arctic and Arctic was probably facilitated by apomixis (Brochmann and Brysting, 2008; Mráz et al., 2009; Hörandl, 2023). In some Arctic plant lineages, diversification appears to have occurred as the result of novel adaptation to colder, more open, and/or wetter environments, for instance in some *Artemisia* lineages, as well as in some *Ranunculus* lineages where previous affinities for wet habitats or tundra vegetation may have primed diversification in the Arctic (Hoffmann et al., 2010; Emadzade et al., 2015).

**Figure 1:**
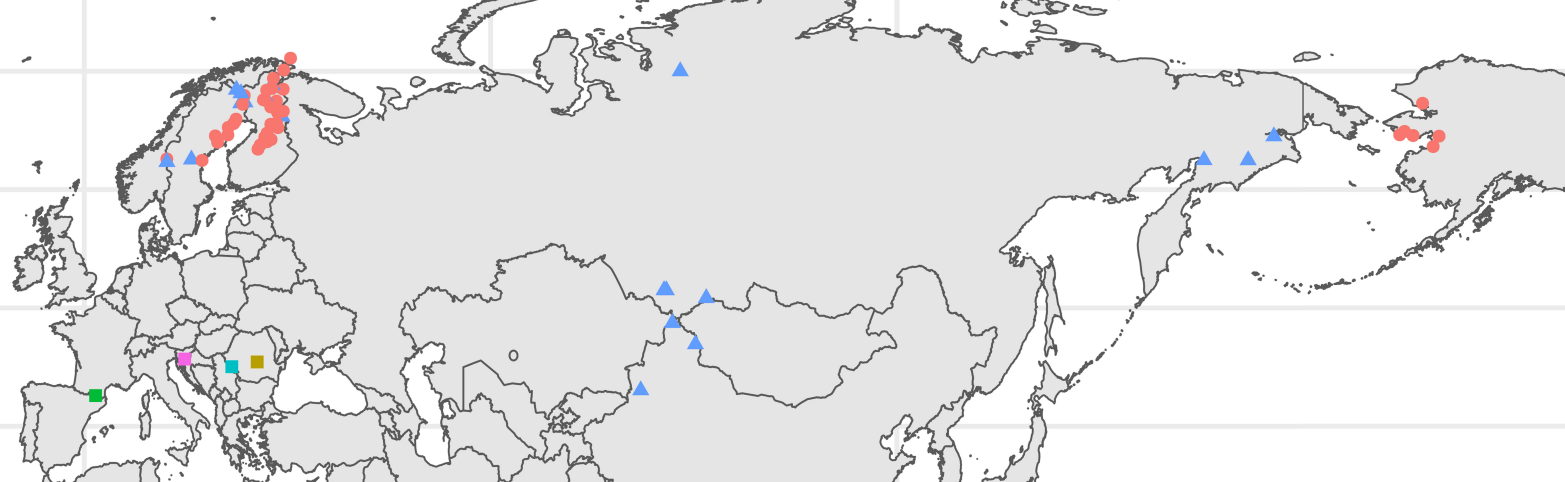
Locations of the individuals sampled. Points are color coded according to taxonomic determination, with *red* corresponding to the *Ranunculus auricomus* morphogroup, *blue* to the *Ranunculus monophyllus* morphogroup, *yellow* to *Ranunculus cassubicifolius*, *green* to *Ranunculus envalirensis*, *turquoise* to *Ranunculus flabellifolius*, and pink to *Ranunculus notabilis*. *Shapes* correspond to key groups, with *dots* indicating the *R. auricomus* morphogroup members, *triangles* representing the *R. monophyllus* morphogroup members, and *squares* indicating the sexual progenitor species. Plot was produced in R using the ggplot2, sf, and ggspatial packages. Full location details are given in **Supplementary Table S1**.

The *Ranunculus auricomus* species complex includes five basal sexually reproducing taxa endemic to areas in Central and Western Europe and hundreds of facultatively asexual morphotypes spread across Eurasia and into the Seward Peninsula, Alaska (**Figure 1**) (Fagerström and Kvist, 1983; Jalas and Suominen, 1989; Ericsson, 1992; Parker, 1999; Krasnoborov, 2000; Kozhevnikov et al., 2019). Owing to periods of hybridization during the last glacial cycles in Europe, hybrid forms are frequent, representing the vast majority of populations in Europe (Tomasello et al., 2020; Karbstein et al., 2022). Hybrid genotypes are typically polyploid, most often tetraploid or hexaploid, and propagate via apomixis to varying degrees (Nogler, 1984; Hörandl, 1998; Barke et al., 2020). Some populations, for example in Southern Europe, seem to be obligate apomicts (Bradican et al., 2023). Morphological diversity in the group is found in the variety of plant heights, basal and stem leaf shapes, petal sizes, and number of stems and basal leaves, among other traits (Marklund, 1961a; Marklund, 1961b; Ericsson, 2001). *Ranunculus monophyllus* Ovcz. represents a reduced form of the more robust morphotypes present in the *R. auricomus* species complex (Ericsson, 2001; Kozhevnikov et al., 2019). In contrast to *R. auricomus*, flowers are typically few and borne on one to several flowering stems per plant (Ericsson, 2001). Basal leaves are similarly present in lower numbers and typically exhibit fewer sinuses than that in *R. auricomus* with up to three lobes (Ericsson, 2001). In addition, a basal leaf sheath is typically present (Ericsson, 2001). *R. monophyllus* and approximately 12 other similar dwarfish taxa have been described as species and summarized as the *R. monophyllus* group (Borchers-Kolb, 1983, Ericsson, 2001) and are largely restricted to higher latitudes and elevations in Europe, but are widely distributed in the range of *R. auricomus* agg. east of the Caucasus and in northern Fennoscandia, on Arctic islands, and in Greenland (Fagerström and Kvist, 1983; Jalas and Suominen, 1989; Parker, 1999; Krasnoborov, 2000; Ericsson, 2001).

In this study, we elucidate the form of reproduction present in the Northern European populations of *R. auricomus* agg., as well as the phylogenetic placement of the hitherto understudied *R. monophyllus* morphogroup and of other populations in Northern Europe, Asia, and Beringia. Furthermore, we incorporate studies of groups of dwarfed and robust forms from boreal Fennoscandia subjected to artificial cold and temperate climate regimes, recording the morphological traits in order to examine the phenotypic plasticity in response to climate. As previous studies would seem to indicate that sexual reproduction is rare in populations present at the margins of this group’s range, we examine whether this extends to Northern Europe where the populations are typically less isolated and more numerous than those in Southern Europe (Ericsson, 2001; Bradican et al., 2023). Although phenotype has previously been the basis for the classification of many of the taxa in this species complex, more recent research has demonstrated in multiple cases that the morphological taxa are often polyphyletic when sequence data are taken into consideration (George et al., 2014; Bradican et al., 2023; Hodač et al., 2023). To date, it is unclear whether the relatively widespread *R. monophyllus* morphogroup represents a distinct lineage, as opposed to the many mosaic-like hybrid networks typical of this group (Karbstein et al., 2022). In the case of hybrid populations, certain geographic trends in the admixture involving several sexual progenitor species have been observed in Europe, proceeding along longitudinal gradients (Karbstein et al., 2022; Bradican et al., 2023). As Europe represents a fraction of the total range of this group, albeit the most well-documented fraction, it remains to be seen whether such trends extend to the Asian and North American representatives of *R. auricomus* agg. Beyond this, it has been suggested that additional hitherto undiscovered or possibly extinct sexual lineages may be present, which may be detectable in populations outside of Central, Southern, and Eastern Europe (Karbstein et al., 2022; Hodač et al., 2023).

## 2 Materials and methods

### 2.1 Collection and sampling

The collection details for the sexual progenitor species utilized here are listed in Tomasello et al. (2020). A total of 36 Fennoscandian populations of *R. auricomus* agg. were collected in the summers of 2021 and 2022. Living plants were kept at the Old Botanical Garden of the University of Göttingen, and herbarium specimens from each population were stored at the Department of Systematics, Biodiversity and Evolution of Plants, Albrecht von-Haller Institute for Plant Sciences, University of Göttingen (herbarium GOET). In addition, material from one population in Alaska (JBAK01) was collected in the summer of 2022, with the herbarium specimens gathered as above. All remaining individuals were sourced from other herbaria due to travel restrictions (**Supplementary Table S1**). See **Supplementary Table S1** for the GPS coordinates and taxonomic determination of all individuals.

### 2.2 Determination of reproductive mode

We utilized an established method for the determination of sexual *versus* apomictic reproduction in individual seeds, dubbed flow cytometry single-seed screening (FCSS), which was developed after Matzk et al. (2000) (Karbstein et al., 2021). Seeds from the Fennoscandian populations were collected from plants either *in situ* or, if undeveloped, in the field after the collection and bagging of developing achenes. The data were gathered as described in Karbstein et al. (2020) through the extraction of nuclei from seed tissue after lysis using a Tissue Lyzer II (Qiagen, Hilden, Germany) and shaking the samples for 10–15 s at 30 Hz for maceration while suspended in a lysis buffer. The solution after maceration was filtered through 30-µm filters to obtain a suspended nuclei solution. The nuclei were stained with DAPI and the solution measured using a CyFlow Ploidy Analyzer (Sysmex, Nordstedt, Germany) and CUBE16 v.1.6 software (Sysmex, Nordstedt, Germany) (Barke et al., 2020). Peaks of the detected genetic material corresponding to the nuclei were marked, and the median sizes of these were inferred as either embryo or endosperm nuclei. The median embryo and endosperm nuclei sizes were then compared to the median nuclei size of somatic tissue from a diploid *Ranunculus cassubicifolius* standard in order to determine ploidy (Barke et al., 2020). The reproduction mode was then inferred via a peak index (PI) metric, which corresponds to the ploidy of the endosperm tissue divided by that of the embryonic tissue. In *R. auricomus* agg., the sexual production of seeds corresponds to a PI between 1.7 and 2, whereas a PI above 2 indicates that the seed was produced through apomixis (Karbstein et al., 2020).

Somatic ploidy was similarly measured, except that the tissue (silica-dried leaf material) was macerated before being added to the lysis buffer, and the peaks of nuclei were interpreted as somatic tissue. When the embryonic tissue was not detectable during FCSS and the endosperm tissue was measured to be more than twice that of the somatic tissue from the mother plant, somatic ploidy was used in lieu of embryo ploidy to obtain a PI metric. The full list of the somatic and seed ploidies is included in **Supplementary Table S1**.

### 2.3 Gathering of sequence data

A modified extraction protocol using the Qiagen Dneasy Plant Mini Kit (Qiagen, Hilden, Germany) and silica-dried leaf or herbarium material was utilized for the extraction of DNA as described in Bradican et al. (2023). Amplified libraries were generated via target enrichment using a bait set designed for *R. auricomus* agg., which consisted of 17,988 probes covering 736 low-copy nuclear regions, as in Tomasello et al. (2020). Details of the pooling concentration are included in **Supplementary Table S1**. Sequences were generated on an Illumina MiSeq platform (Illumina, San Diego, CA, USA) in two paired-end runs. The sequences of sexual progenitor species have been generated previously in Tomasello et al. (2020), from which we included representatives of each lineage. Here, we generated the sequence data for 79 individuals. Of these, 17 populations including a total of 23 individuals were determined to fall into the *R. monophyllus* morphogroup (**Supplementary Table S1**). The remaining 38 populations, which included 56 individuals, were determined to fall into a broader *R. auricomus* morphogroup (**Supplementary Table S1**).

### 2.4 Phylogenomic analyses

We utilized the HybPhyloMaker pipeline in order to process raw reads for adapter sequences and low-quality reads, retaining filtered reads after running “HybPhyloMaker1_rawprocess” (Fér and Schmickl, 2018). The filtered reads were then run through the HybPiper pipeline in order to gather supercontig sequences (Johnson et al., 2016; Bradican et al., 2023). The HybPiper functions “assemble” and “intronerate” were used, incorporating bwa for mapping (Li and Durbin, 2009; Johnson et al., 2016). A single nucleotide polymorphism (SNP) dataset was constructed using HybPiper supercontigs as a reference (see https://github.com/lindsawi/HybSeq-SNP-Extraction) by mapping the trimmed reads utilizing “bwa mem.” Variant sites were identified using HaplotypeCaller in GATK (Li and Durbin, 2009; McKenna et al., 2010; Danecek et al., 2021). The GVCF files were then combined and called using “CombineGVCF” and “GenotypeGVCF.” Subsequently, variants were selected with “SelectVariants” and filtered with “VariantFiltration” using a hard filter and the following expression: – filterExpression “QD < 5.0 || FS > 60.0 || MQ < 40.0 || MQRankSum < −12.5 || ReadPosRankSum < −8.0” (McKenna et al., 2010; Cooper et al., 2023). We filtered the SNPs using PLINK, executing –vcf-filter –vcf V87107.snp.filtered.nodot.vcf –allow-extra-chr –recode –make-bed –geno –const-fid –out V87107, utilizing the individual with the highest read count and depth (V87107) as a reference (Purcell et al., 2007). The filtered VCF matrix was then imported into R and converted into a genind object for principal component analysis using the dudi.pca function without scaling in the *adegenet* package (Jombart, 2008).

For use in SplitsTree v. 4.17.1, the filtered SNP matrix produced from the above-mentioned method was converted into Nexus format in R using vcf2phylip (Huson and Bryant, 2006; Ortiz, 2019). A phylogenetic network was produced on a filtered SNP dataset with 27,586 polymorphic sites. The clusters in the network were assessed via 1,000 bootstrap replicates using uncorrected *p* character transformation and NeighborNet distance transformation with a lambda frac of 1.0 and ordinary least squares variance (Bryant, 2003).

The HybPhaser pipeline was utilized for clade association analysis (Nauheimer et al., 2021). The clade association values were plotted next to a phylogenetic tree calculated in RAxML-NG from the PHYLIP matrix detailed above (Kozlov et al., 2019). This was done in R using the “phylo.heatmap” function in the *phytools* package (Revell, 2012). For the full parameters used in HybPhaser and RAxML-NG, see **Supplementary Table S2**.

### 2.5 Climate chamber experiments

Living plants were used in 2 years (2022 and 2023) for climate chamber experiments using two regineering climate chambers (regineering GmbH, Pollenfeld, Bavaria, Germany). The plants were divided into four groups: robust plants in a cold and a temperate treatment and dwarf plants in a cold and a temperate treatment. The grouping into “dwarf” and “robust” followed phenotypes as observed in the field, following the descriptions of Ericsson (2001) for grade 4 for “dwarf” and grades 1–3 for “robust.” The climate regimes consisted of a simulated spring and early summer, with temperatures staying above 6°C and the humidity kept at 65% relative humidity. The temperatures and light durations/intensities were stepped up at 2-week intervals. Temperate treatment ranged from 10°C to 20°C, and the light intensity and durations were comparable to open conditions in Central Europe. In contrast, the cold treatment was kept at 67% of the light intensity, but with a longer light duration, comparable to northern Sweden, and with temperatures ranging from 6°C to 10°C (see **Supplementary Table S1** for the full climate regimes). In 2022, the measurements could be taken from 26 robust plants and 16 dwarf plants (**Supplementary Table S1**). In 2023, the measurements were taken from 42 robust plants and 36 dwarf plants (**Supplementary Table S1**). All plants were collected from the field in northern Finland, Norway, and Sweden. After treatments, measurements were taken for stature, number of basal leaves, number of stems, number of flowers, and leaf surface area. Stature was measured directly in centimeters, while organ numbers were counted. The leaf surface area was measured by taking pictures of the flattened leaves with a metric ruler, without detaching the leaves from the plants. Images were then processed in ImageJ by establishing a known length of 1 mm from the metric ruler, then tracing around the edge of leaves and calculating the surface area (Schneider et al., 2012).

The measurement values were tested for normality with the Shapiro–Wilk test and for homogeneity of variance with Levene’s test. Outliers were removed after being identified using the Grubbs test function in the outlier package in R (Komsta, 2006). Multi-way ANOVA and Tukey’s honest comparison of means were utilized to examine differences between groups. All data are included in **Supplementary Table S1**, and the statistical results are listed in **Supplementary Table S2** (Fox and Weisberg, 2019; R Core Team, 2022).

## 3 Results

### 3.1 Apomixis in northern Fennoscandian populations

The somatic ploidy of the *R. auricomus* agg. populations in northern Fennoscandia was determined to be tetraploid or hexaploid (**Table 1**; **Supplementary Table S1**). We observed a strong tendency toward apomictic propagation in the populations examined (**Table 1**). Sexual reproduction was observed in only three of the 33 populations investigated, with overall low percentages of sex in these populations (ranging from 2.6% to 7.7% sexual) (**Table 1**).

**Table 1:** Percentage of sexual seeds in 33 populations of *Ranunculus auricomus* agg. collected in northern Fennoscandia.

Population ID	Somatic ploidy	Percentage sexual reproduction	No. of seeds
SE2	4*x*	0	24
SE3	6*x*	0	25
SE4	6*x*	7.7	13
SE5	4*x*	0	20
SE6	4*x*	0	27
SE7	4*x*	0	24
SE8	4*x*	0	26
SE9	4*x*	0	17
SE10	4*x*	0	30
SE11	4*x*	0	27
SE12	4*x*	0	13
SE13	4*x*	0	38
SE14	4*x*	0	28
SE15	4*x* and 6*x*	0	20
SE16	6*x*	0	31
SE17	6*x*	0	28
SE18	4*x*	0	28
SE19	4*x*	0	29
JB020	4*x*	0	39
JB022	4*x*	0	20
JB025	4*x*	4.2	24
JB027	4*x* and 6*x*	0	15
JB029	4*x*	0	40
JB030	4*x*	0	17
JB031	4*x*	0	10
JB032	4*x*	0	57
JB033	4*x*	2.6	38
JB034	4*x*	0	27
JB035	4*x*	0	53
JB036	4*x*	0	50

Sexual versus apomictic reproduction was determined using flow cytometry single-seed screening. Listed are the somatic ploidy levels found in each population. Full values per population, ploidy, and individuals are listed in **Supplementary Table S1**.

### 3.2 Network analysis

**Figure 2:**
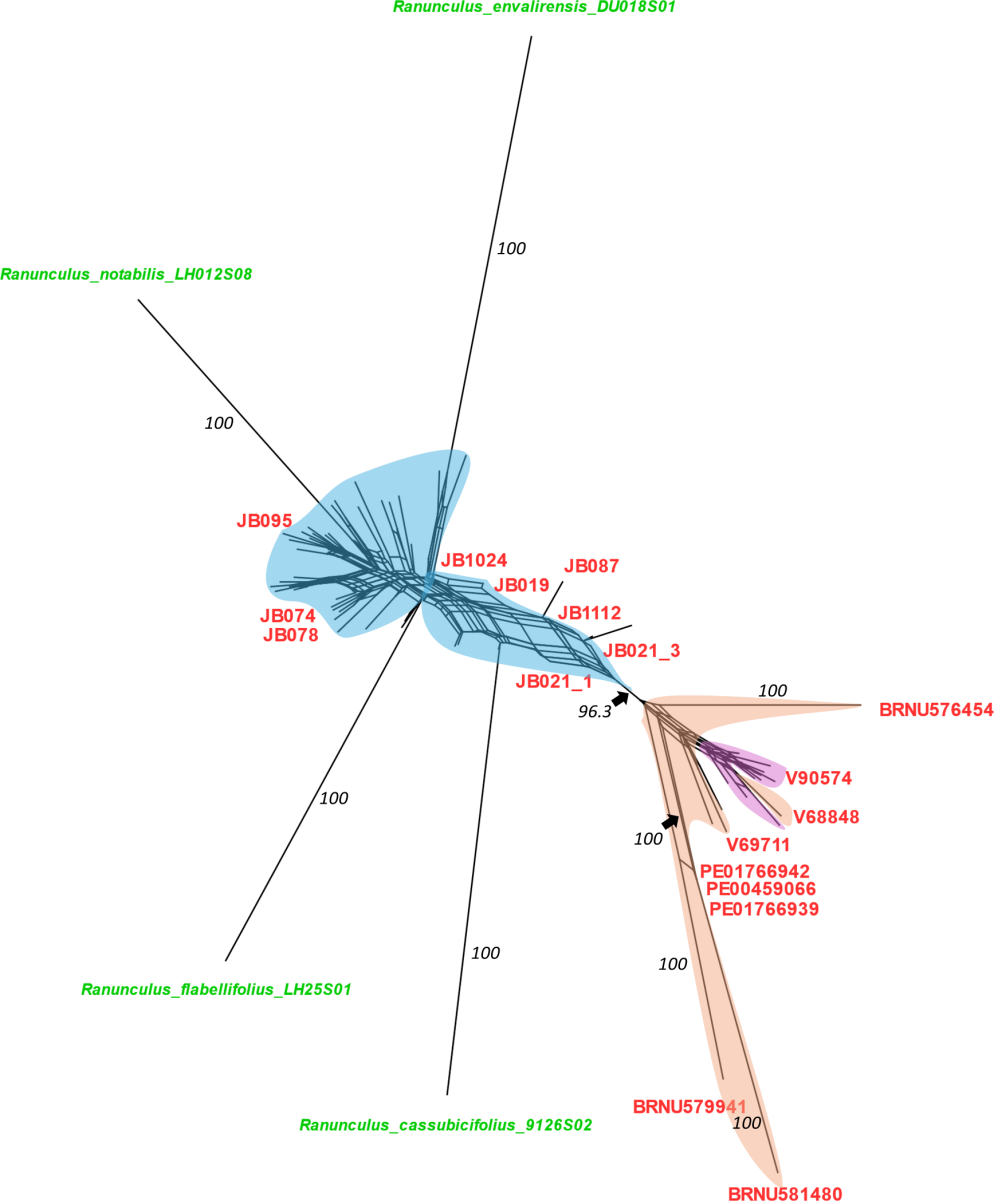
SplitsTree NeighborNet network of a filtered SNP matrix for the Fennoscandian, Central Asian, Beringian *Ranunculus auricomus* members, and four sexual progenitor species (i.e., *Ranunculus cassubicifolius*, *Ranunculus envalirensis*, *Ranunculus flabellifolius*, and *Ranunculus notabilis*). Sections of the network are color coded according to the geographic location of individual clades: *blue*, Fennoscandia; *light orange*, Central Asia; *pink*, Beringia. *Tips* are *color coded* according to taxonomic determination, with *red* corresponding to *Ranunculus monophyllus*, *green* to the sexual progenitor taxa, and *black* to *R. auricomus* hybrids (IDs not shown). Bootstrap values for the major clusters and sexual progenitor taxa are indicated in *black*.

A phylogenetic network was produced in SplitsTree4 from a filtered SNP dataset that included representatives of four diploid sexual progenitor species (i.e., *R. cassubicifolius*, *Ranunculus envalirensis*, *Ranunculus flabellifolius*, and *Ranunculus notabilis*), representatives of 36 Fennoscandian populations, and 15 Asian and North American populations (**Figure 2**). A primary split (BS support = 96.3) was observed between the Fennoscandian individuals and all of the Asian and North American populations (**Figure 2**). Within the latter, the Beringian individuals largely clustered together, except for V129074, being nested among the Central Asian individuals (**Figure 2**). Comparison of the individuals determined as *R. monophyllus* to the other *R. auricomus* agg. individuals revealed no clear pattern or distinction, with *R. monophyllus* being present across the network (**Figure 2**).

### 3.3 Clade association

**Figure 3:**
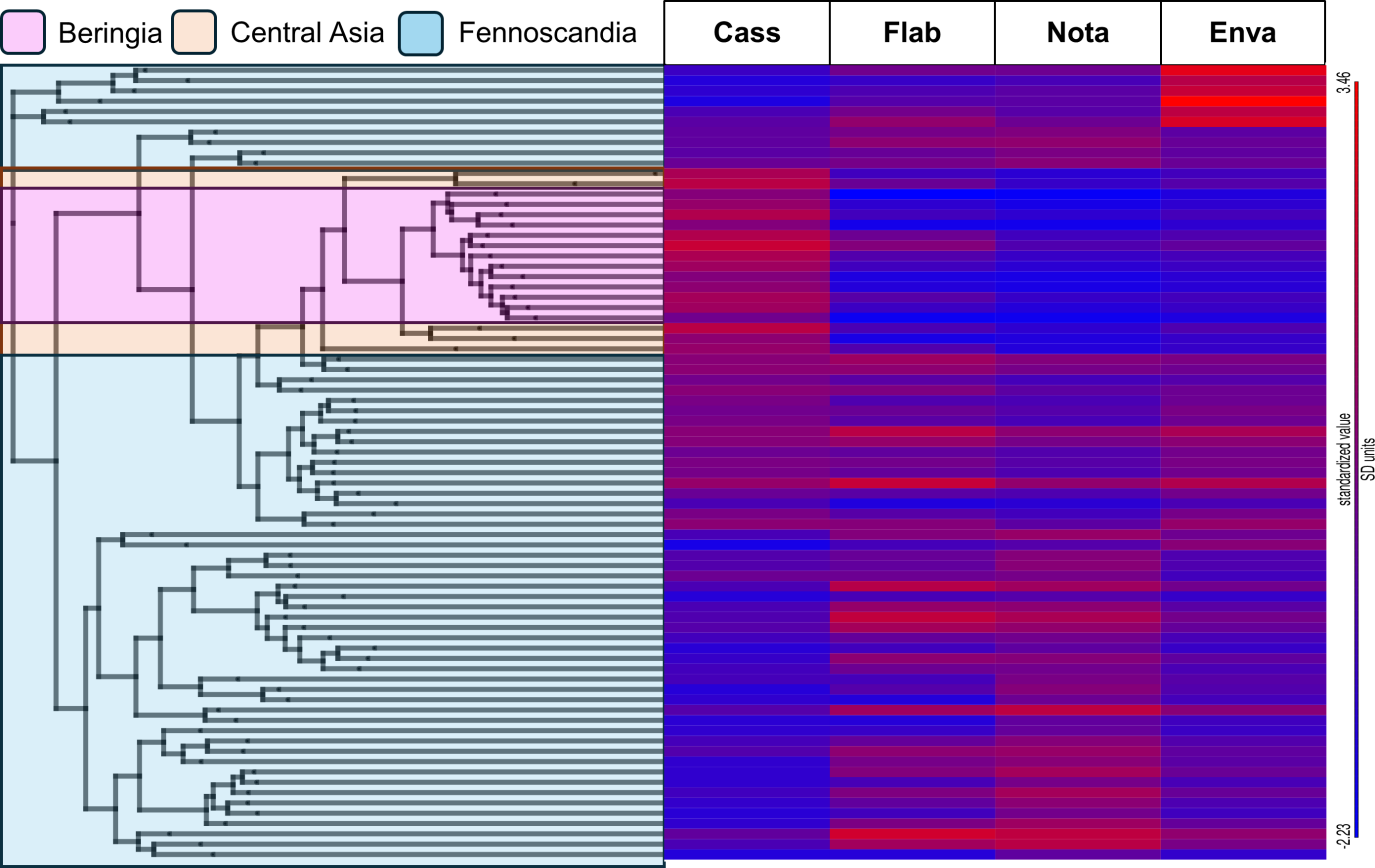
Clade association heatmap representing admixture to *Ranunculus auricomus* agg. individuals from basal sexual progenitor members. A RAxML-NG phylogeny is displayed to the *right*, computed from a filtered SNP dataset, and color coded according to the geographic location of individuals: *pink*, Beringia; *light orange*, Central Asia; *light blue*, Fennoscandia. A standardized clade association heatmap (*red* indicates higher association and *blue* indicates lower association) is displayed to the *right*, with *rows* corresponding to individual tips of the RAxML phylogeny and *columns* representing sexual progenitor species: *Cass*., *Ranunculus cassubicifolius*; *Flab*., *Ranunculus flabellifolius*; *Nota*., *Ranunculus notabilis*; *Enva*., *Ranunculus envalirensis*. Values were calculated using HybPhaser. See **Supplementary Table S1** for full clade association values.

Detection of similarity between putative *R. auricomus* agg. hybrids and sexual progenitor taxa suggested a hybrid origin for most individuals (**Figure 3**). All Asian and Alaskan individuals tended toward a higher similarity to *R. cassubicifolius* (**Figure 3**, highlighted in light orange and pink). Fennoscandian individuals showed a broader mixture, with some western Swedish individuals having high admixture from *R. envalirensis* (**Figure 3**, top six rows).

### 3.4 Principal component analysis

**Figure 4:**
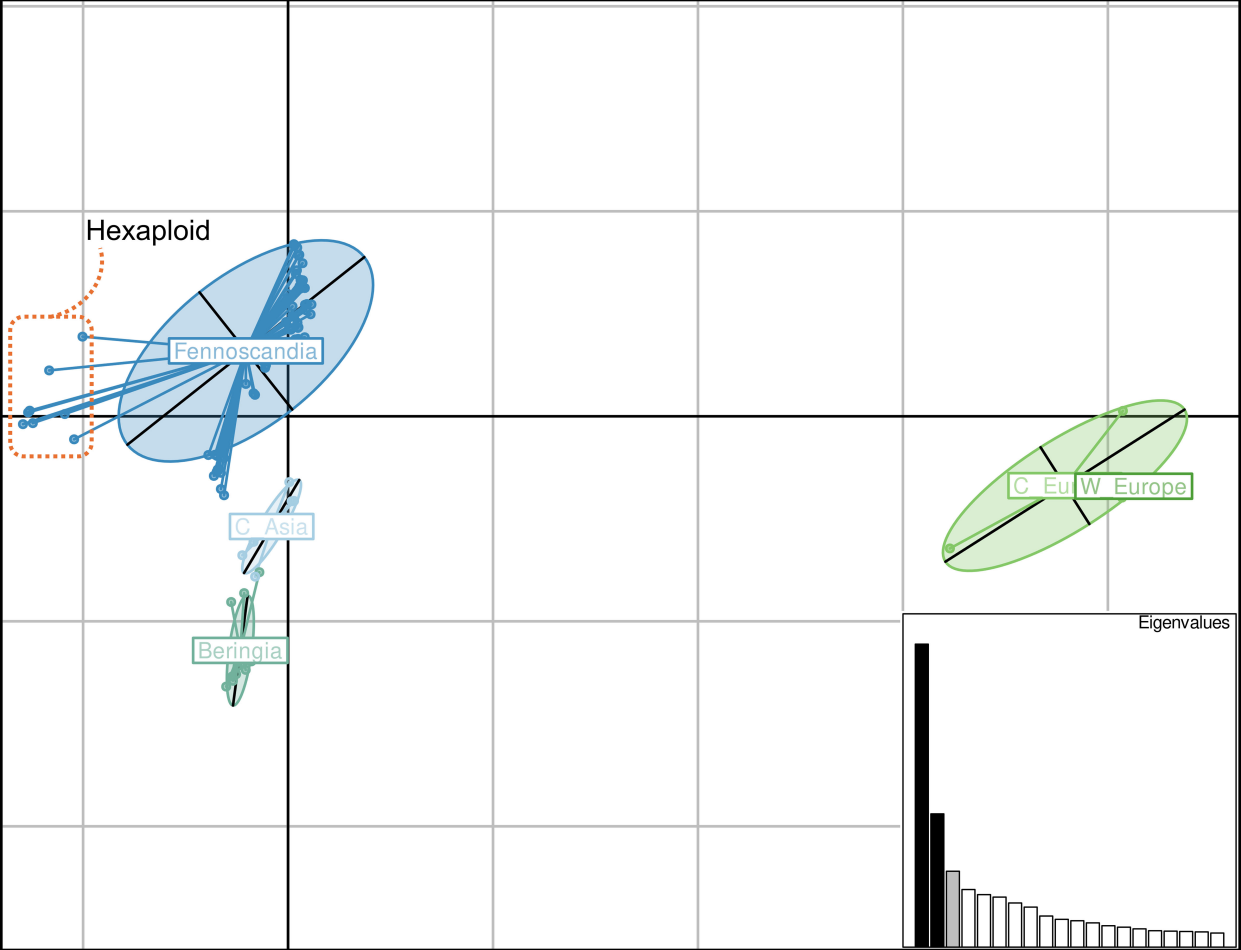
Principal coordinate analysis of a filtered SNP dataset for all putative hybrid individuals and sexual progenitor species. *X*-axis: principal component 1, representing 21.14% of variation; *Y*-axis: principal component 2, representing 9.29% of variation. Groupings by geographic location are indicated by *lines connecting points* and an *ellipse*. Individuals are segregated according to the following geographic locations: Western (*W Europe*) and Central (*C Eu*) Europe for the four sexual progenitor species, Fennoscandia for putative hybrids, Central Asia (*C Asia*) for putative hybrids, and Beringia for putative hybrids. Known hexaploid individuals are bordered by the *orange dashed box*. Eigenvalues are plotted as a bar plot in the *bottom right corner*. Values were calculated in R using the adegenet package.

Plotting of the first two principal components of a filtered SNP dataset showed some differentiation between geographic origins (**Figure 4**). Except for the sexual progenitor taxa (right, labeled as W Europe and C Europe), the Fennoscandian individuals exhibited the highest variation in differences within a geographic group (**Figure 4**). Some separation was observed between the Fennoscandian, the Central Asian, and the Beringian individuals, with some overlap between the latter two (**Figure 4**). Those most leftward on the *x*-axis in the Fennoscandian group are all known hexaploid individuals in the dataset (**Figure 4**).

### 3.5 Climate chamber experiments

**Figure 5:**
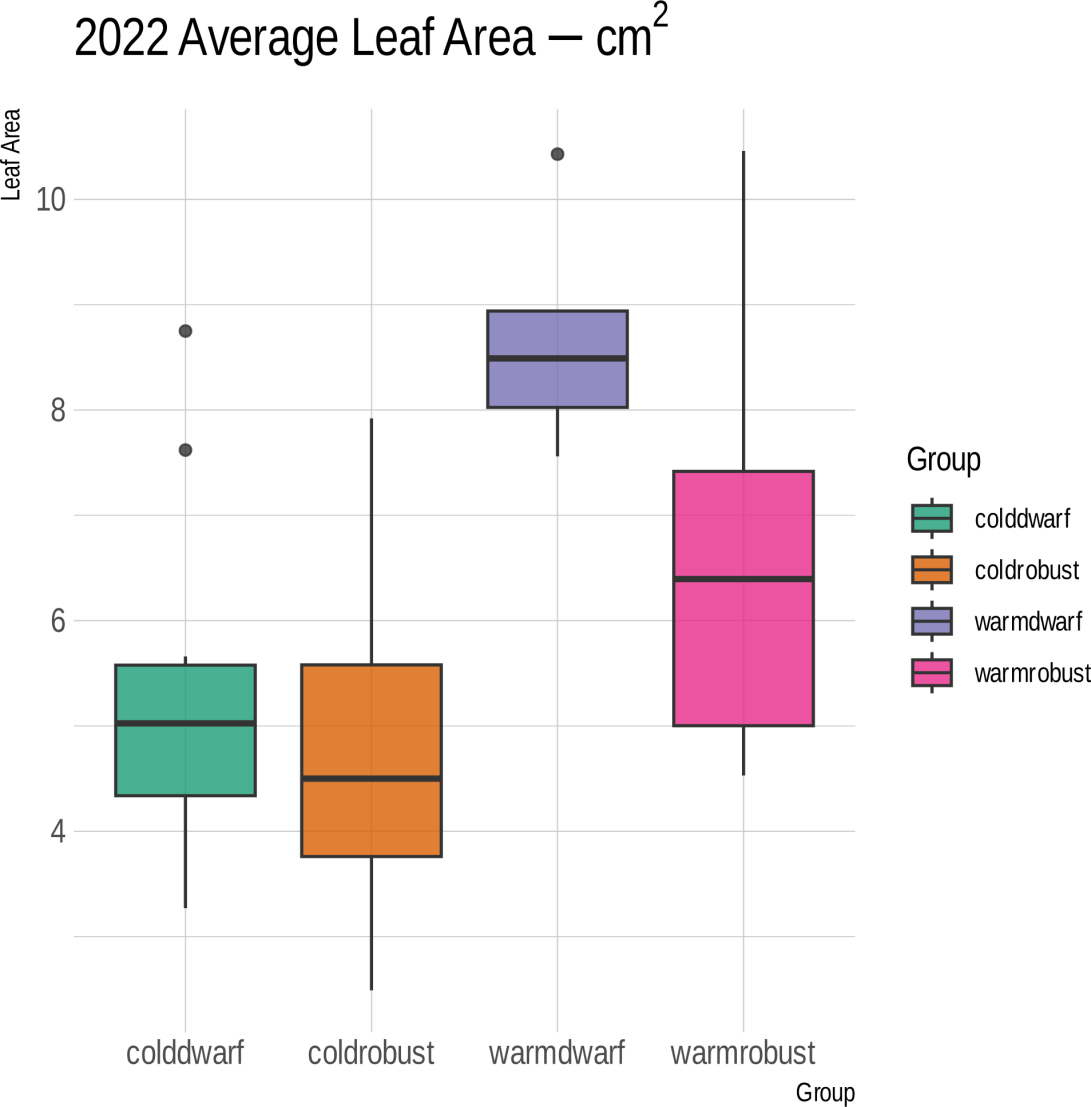
Box plot of the leaf sizes in square centimeters (*y*-axis) between morphogroups (Dwarf or Robust) and climate treatments (Warm or Cold) during the 2022 trial of climate experiments. *Colors* and *boxes* correspond to either cold-treated dwarf or robust plants (*coldwarf* and *coldrobust*, respectively) or warm-treated dwarf or robust plants (*warmdwarf* and *warmrobust*, respectively). The dwarf plants under a warm treatment had significantly larger leaves than the dwarf plants in a cold treatment (*lavender vs*. *pink box*, *p* = 0.0032).

Over the course of 2 years, few differences were observed between the corresponding morphogroups subjected to differing climate regimes. A significantly larger leaf area in the dwarf plants under a temperate regime was observed during the 2022 trial (*p* = 0.0032) (**Figure 5**). In addition, in 2023, the dwarf plants under a temperate treatment exhibited a lower height than the dwarf plants under a cold treatment (*p* = 0.0027) (**Supplementary Table S2**). All other characteristics did not show significant lability in response to climate (**Supplementary Table S2**).

## 4 Discussion

The reproductive data from Northern Europe reinforced previous observations of a predominantly apomictic propagation in the marginal areas of the range of *R. auricomus* agg (Bradican et al., 2023). In contrast to the Southern European populations, which are relegated to mesic, shady, and relatively high elevation habitats, the populations of *R. auricomus* agg. in northern Fennoscandia tolerate a wider range of conditions, inhabiting mountain birch and spruce forests, natural meadows, pasture meadows, and disturbed sites such as roadsides, ditches, parks, and campgrounds (Marklund, 1961a; Marklund, 1961b; Ericsson, 2001). Occurrence in a greater variety of habitats, as well as a more contiguous distribution, would suggest a less isolated and prolific status of the Northern European *R. auricomus* agg. compared with the Southern European populations (Dunkel, 2021; Dunkel, 2015; Dunkel, 2010; Jalas and Suominen, 1989). Similar to populations at the southern margins, apomixis remains common, suggesting a certain stability in the traits underlying a tendency toward the apomictic mode. As previously hypothesized, this mode of reproduction could prove advantageous in the rapid spread into and the occupation of newly open habitats, congruent with a geographical parthenogenesis scenario (Tilquin and Kokko, 2016; Karbstein et al., 2021; Hörandl, 2022). Factors relating to the reversibility of this trait under stable conditions remain to be thoroughly investigated, and although obligate asexual propagation has been theorized to be deleterious in the long term, advantageous traits gained through heterozygosity as a result of hybridization events may be stabilized through asexual propagation (Muller, 1964; Hörandl et al., 2020).

Examination of the individual representatives of the *R. monophyllus* group across Eurasia revealed significant differentiation but no clear grouping of the samples of this taxon. This mirrored previous investigations into other taxa within *R. auricomus* agg., whereby habit did not correspond to genetic relatedness (Karbstein et al., 2022; Bradican et al., 2023; Hodač et al., 2023). This is likely due to factors facilitating significant phenotypic plasticity in the group, perhaps further facilitated by the history of hybridization and concurrent polyploidy (Hodač et al., 2023). The patchwork genetic makeup of many *R. auricomus* agg. consisting of subgenomic elements from divergent progenitor species lends itself to the diverse expression of and the selection for favorable traits (Karbstein et al., 2022; Hodač et al., 2023). Environmental pressures consistent across the range of *R. monophyllus* include a trend toward colder temperatures, a factor previously associated with the presence of dwarf or cushion forms of widespread lineages (Hijmans et al., 2005; Körner, 2021). The climate chamber experiments detailed here, however, suggested that a hypothesized dwarfing response to climate is not quickly inducible, more likely representing either long-term adaptation to a colder climate or local adaptation to other factors such as edaphic conditions and wind exposure. This contrasts with the alpine plant *Ranunculus kuepferi*, a species with autotetraploid apomictic populations, for which similar temperature treatments revealed phenotypic plasticity in vegetative traits (Syngelaki et al., 2020). Considering this, the morphology associated with *R. monophyllus* may represent multiple separate adaptive expressions of hybrid genotypes, possibly including significant epigenetic control (Lloyd and Lister, 2022). It must be noted, however, that we did not incorporate here the alpine Central European taxa previously included in the *R. monophyllus* group, such as *Ranunculus allemanni*, *Ranunculus melzeri*, and *Ranunculus braun-blanquetii* (Hörandl and Gutermann, 1998). Thus, it remains an open question whether different evolutionary histories or ecological differences between Arctic and temperate alpine conditions play a role in phenotypic plasticity. Taking hybrid origin and non-monophyly into account, it is suggested that the microspecies described under the *R. monophyllus* group may better be recognized as nothotaxa, as suggested for the other apomictic taxa of the *R. auricomus* complex (Karbstein et al., 2022; Bradican et al., 2023; Hodač et al., 2023). Considering the findings listed here and in earlier research, an integrative approach to taxonomy in *R. auricomus* agg. examining populations and/or taxa on a case-by-case basis utilizing reproductive and sequence data is necessary for classification (Hörandl, 2022).

When examining the similarities between genotypes across northern Eurasia, certain groups become evident. This concerns primarily as differentiation according to geographic location, roughly segregating into Fennoscandian, Central Asian, and Beringian groups, echoing previous findings in Southern and Central Europe (Karbstein et al., 2022; Bradican et al., 2023). Reinforcing the observations on *R. monophyllus* above, this is particularly striking given the taxonomic and morphological complexity present in Fennoscandia (Marklund, 1961a; Marklund, 1961b; Ericsson, 1992; Ericsson, 2001). Taking into account the likelihood of a rapid expansion into these regions, the differences observed may reflect observable post-hybridization genome evolution (Eroukhmanoff et al., 2013). Some sources and observation data suggest that *R. monophyllus* may be more isolated in Siberia and the Russian Far East, which may also contribute to their differentiation from European *R. auricomus* agg (Kozhevnikov et al., 2019; GBIF.org, 2024). If the trend observed in Europe continues to the east, and Asian/Alaskan populations are also largely apomictic, further isolation and geographic differentiation is likely. Despite genetic similarity, it cannot be concluded that the geographic groups found here correspond to distinct hybridization events. Indeed, as discussed further below, different levels of admixture from predecessor species are detected within geographic groups.

Taking into account the likely hybrid origin of the populations examined here, clade association revealed diversity in the admixture from sexual progenitor species to Northern Eurasian *R. auricomus* agg. Almost all individuals show evidence of the presence of all known sexual progenitor genomes in their respective genotypes. Mirroring findings in Southern Europe, the southwestern progenitor *R. envalirensis* from the Pyrenees and Massif Central appears to be represented more in the hybrid genomes present in western Sweden. Possibly, the contact zone that may have formed in the southwestern areas of Europe where *R. envalirensis* was present during the genesis of hybrid genotypes led to expansion along more western longitudes both southward and northward. With lower sea levels and the fluctuating presence of land connecting Western Europe and the Fennoscandian Peninsula following the last glacial maximum, further investigation of the Northwestern European *R. auricomus* agg. might help reveal the expansion routes (Björck, 1995; Paus et al., 2023). Land-based migration corridors bridging modern-day Denmark and Sweden would have been most hospitable to *R. auricomus* agg. from circa 13.1–12.7 ka BP and 12.1–10.3 ka BP due to the presence of solid sediment bridges spanning the Baltic Ice Lake (Björck, 1995; Herman et al., 2014). In Asia and Alaska, the Central to Eastern European *R. cassubicifolius* appears to be the predominant contributor to hybrid genotypes. This again would theoretically align with the likely expansion routes, given the more eastern range of *R. cassubicifolius*. However, we cannot rule out that one or more unknown sexual progenitors contributed to the origins of the populations in Asia, Beringia, and Alaska. As we do not yet have reproductive data from these areas, progenitors can be only theoretically postulated.

## Data availability statement

The data presented in the study are deposited in the Sequence Read Archive repository, accession number PRJNA1097346.

## Author contributions

JB: Data curation, Formal analysis, Investigation, Writing – original draft, Writing – review & editing. ST: Investigation, Methodology, Writing – original draft, Writing – review & editing. JV: Data curation, Formal analysis, Investigation, Writing – original draft, Writing – review & editing. EH: Conceptualization, Funding acquisition, Project administration, Resources, Supervision, Writing – original draft, Writing – review & editing.

## Supplementary material

Supplementary Table 1 contains tables including flow cytometry data, detailed information on individuals used in this study, sequence data filtering metrics, clade association values, morphological data, and climate chamber parameters.

Supplementary Table 2 contains details on phylogenetic methods and statistical results of climate chamber experiments.
