# Supplementary material for: Converging forms: an examination of sub-Arctic, circumarctic, and Central Asian Ranunculus auricomus agg. populations
Source: Front Plant Sci. 2024 Jun 17;15:1415059. doi: 10.3389/fpls.2024.1415059 (PMC11215153; doi:10.3389/fpls.2024.1415059)
Supplement: Supplementary Table 2 — contains details on phylogenetic methods and statistical results of climate chamber experiments. [file Table_2.docx]

**Supplemental**

Phylogenetics

**HybPhaser Configuration**

Used to estimate admixture from progenitor species to putative hybrids

###############################

### Part 1: SNP Assessment ###

###############################

name_for_dataset_optimization_subset = "mono_1"

# missing data

remove_samples_with_less_than_this_propotion_of_loci_recovered = 0.6

remove_samples_with_less_than_this_propotion_of_target_sequence_length_recovered = 0.6

remove_loci_with_less_than_this_propotion_of_samples_recovered = 0.75

remove_loci_with_less_than_this_propotion_of_target_sequence_length_recovered = 0.75

# Paralogs

remove_loci_for_all_samples_with_more_than_this_mean_proportion_of_SNPs = "none" # any number between 0 and 1, "none" or "outliers"

file_with_putative_paralogs_to_remove_for_all_samples = ""

remove_outlier_loci_for_each_sample = "no"

##################################

### Part 2: Clade Association ###

##################################

# set variables to determine thresholds for the dataset optimization and run the script from the main script

path_to_clade_association_folder = "~/Desktop/John/HybPhaser/HybPhaser/out_mono_1/04_clade_association"

csv_file_with_clade_reference_names = "~/Desktop/John/HybPhaser/HybPhaser/clade_references_4.csv"

path_to_reference_sequences = "~/Desktop/John/HybPhaser/HybPhaser/out_mono_1/03_sequence_lists_mono_1/samples_contigs"

path_to_read_files_cladeassociation = "~/Desktop/John/HybPhaser/HybPhaser/out_mono_1/mapped_reads"

read_type_cladeassociation = "single-end"

ID_read_pair1 = ""

ID_read_pair2 = ""

file_with_samples_included = "~/Desktop/John/HybPhaser/HybPhaser/monophyllus_nameslist2nooutgroups.txt"

path_to_bbmap = "/home/hobel2/Software/BBMap_39.01/bbmap"

no_of_threads_clade_association = "auto"

run_clade_association_mapping_in_R = "no"

java_memory_usage_clade_association = "" # e.g. 2G (for 2GB) needed when java -Xmx error comes up. should be max. 85% of physical memory

**Raxml-NG Configuration**

Used to visualize bifurcating tree in order to display heatmap

raxml-ng –all –msa snp_matrix.phy –model GTR+G –seed 2 –bs-metric tbe

**Principal Component Analysis**

See below for workflow in Rstudio using adegenet, used for principal component analysis

### vcf to genind

#import vcf

ra_vcf2 <- read.vcfR("JB009_noout.snp.filtered.nocall.vcf")

#extract genotype data from vcf to a matrix

genotype <- extract.gt(ra_vcf2)

# Transpose the genotype matrix

genotype_matrix_transposed <- t(genotype)

# Convert the transposed genotype matrix to a genind object

genind_object <- df2genind(genotype_matrix_transposed, sep = "\t")

# Check the structure of the genind object

str(genind_object)

# Add ploidy

rownames(ra_ploidy) <- ra_ploidy[,1]

ra_p <- subset(ra_ploidy, select = -c(1))

ploidy_values <- as.integer(ra_p$ploidy)

ploidy(genind_object) <- ploidy_values

# Add pop data

pop_dat <- as.matrix(pop_dat)

rownames(pop_dat) <- pop_dat[,1]

pop_dat <- subset(pop_dat, select = -c(1))

pop(genind_object) <- pop_dat

# Add realm data

pop(genind_object) <- ra_dat$realm

#no scaling basic PCA

sum(is.na(genind_object$tab))

genind_no_NA <- tab(genind_object, freq = TRUE, NA.method = "mean")

gi_pca1<- dudi.pca(df = genind_no_NA, scale = FALSE, scannf = FALSE, nf = 3)

barplot(gi_pca1$eig[1:50], main = "PCA eigenvalues", col = heat.colors(50))

Climate Chamber Morphometric Statistics and Plots

**2022**

Outliers were identified and removed from natural log transformed group (cold dwarf, robust warm, etc.) datasets using the Grubbs Test (implemented in Rstudio using the grubbs.test function in the outliers package). Both single and double outliers on either end were tested for, as well as opposite outliers.

*Plant Height*

*
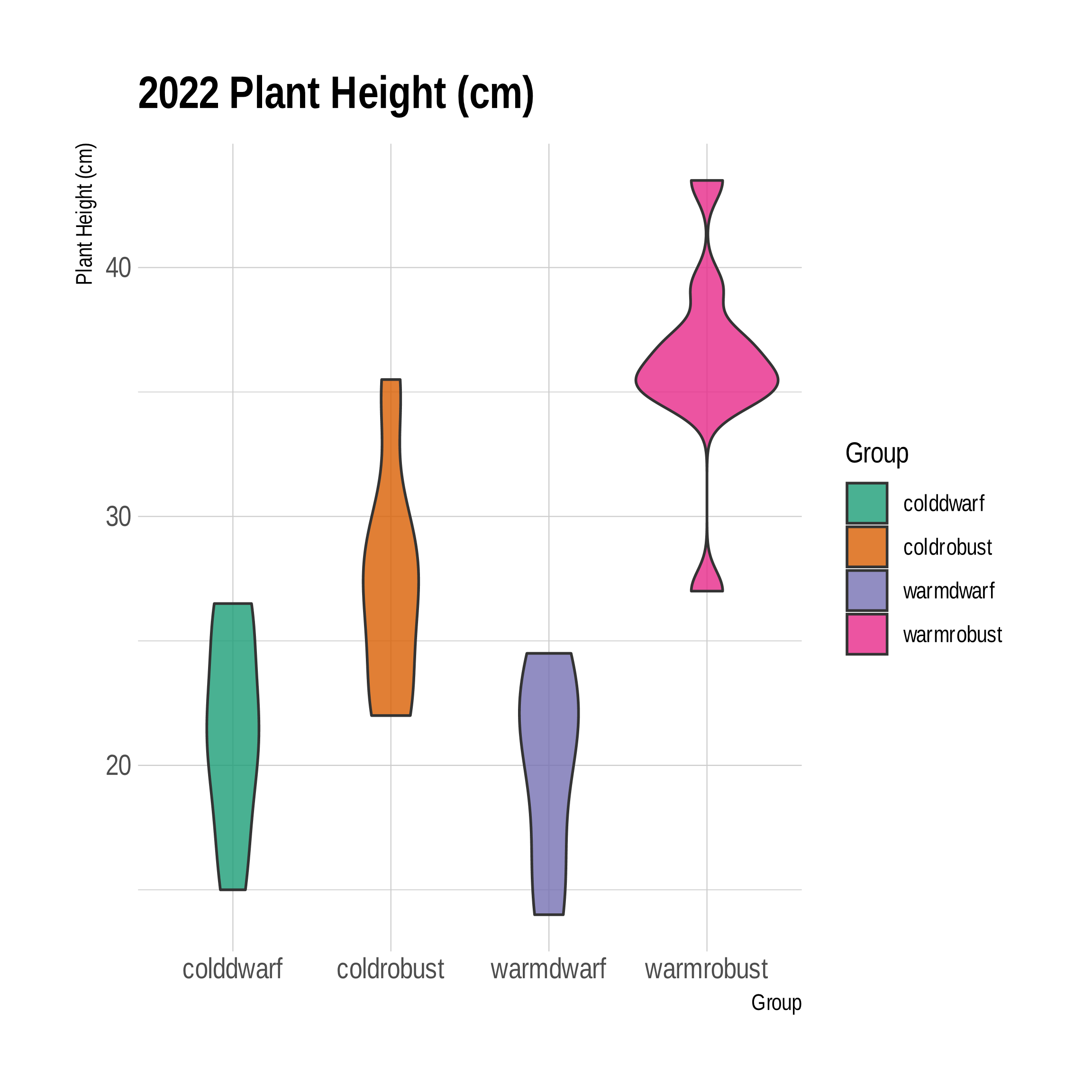
*

***Shapiro Wilk***

Shapiro-Wilk normality test

data: a_height$HEIGHT_L

W = 0.96067, p-value = 0.1878

***Levene***

> leveneTest(a_height$HEIGHT_L, a_height$Morph)

Levene's Test for Homogeneity of Variance (center = median)

Df F value Pr(>F)

group 1 6.1168 **0.01811 ***

37

---

Signif. codes: 0 ‘***’ 0.001 ‘**’ 0.01 ‘*’ 0.05 ‘.’ 0.1 ‘ ’ 1

> leveneTest(a_height$HEIGHT_L, a_height$Treatment)

Levene's Test for Homogeneity of Variance (center = median)

Df F value Pr(>F)

group 1 4.2446 **0.04646 ***

37

---

Signif. codes: 0 ‘***’ 0.001 ‘**’ 0.01 ‘*’ 0.05 ‘.’ 0.1 ‘ ’ 1

> leveneTest(a_height$HEIGHT_L, a_height$Ploidy)

Levene's Test for Homogeneity of Variance (center = median)

Df F value Pr(>F)

group 2 2.0379 0.1451

36

***AO******V***

Df Sum Sq Mean Sq F value Pr(>F)

Morph 1 982.7 982.7 51.026 **4.99e-08 *****

Treatment 1 207.3 207.3 10.764  **0.00256 ****

Ploidy 2 13.5 6.7 0.350 0.70752

Morph:Treatment 1 223.6 223.6 11.610 **0.00183 ****

Morph:Ploidy 1 0.1 0.1 0.004 0.94975

Treatment:Ploidy 1 4.9 4.9 0.253 0.61839

Residuals 31 597.0 19.3

---

Signif. codes: 0 ‘***’ 0.001 ‘**’ 0.01 ‘*’ 0.05 ‘.’ 0.1 ‘ ’ 1

***Tukey HSD***

> TukeyHSD(aovheight)

Tukey multiple comparisons of means

95% family-wise confidence level

Fit: aov(formula = HEIGHT_L ~ (Morph + Treatment + Ploidy)^2, data = a_height)

$Morph

diff lwr upr p adj

robust-dwarf 10.20516 7.291427 13.1189 **0**

$Treatment

diff lwr upr p adj

warm-cold 4.678767 1.765031 7.592504 **0.0026024**

$Ploidy

diff lwr upr p adj

4-3 3.7059297 -7.317700 14.729559 0.6891012

6-3 3.4871511 -7.692850 14.667152 0.7253230

6-4 -0.2187786 -3.851084 3.413527 0.9879628

$`Morph:Treatment`

diff lwr upr p adj

robust:cold-dwarf:cold 5.993131 0.9832324 11.003029 0.0141261

dwarf:warm-dwarf:cold -1.229919 -7.3805692 4.920731 0.9478071

robust:warm-dwarf:cold 14.620000 9.2933808 19.946619 **0.0000001**

dwarf:warm-robust:cold -7.223050 -13.1015447 -1.344555 0.0113244

robust:warm-robust:cold 8.626869 3.6169713 13.636768 **0.0003050**

robust:warm-dwarf:warm 15.849919 9.6992691 22.000569 **0.0000004**

$`Morph:Ploidy`

diff lwr upr p adj

robust:3-dwarf:3 NA NA NA NA

dwarf:4-dwarf:3 NA NA NA NA

robust:4-dwarf:3 NA NA NA NA

dwarf:6-dwarf:3 NA NA NA NA

robust:6-dwarf:3 NA NA NA NA

dwarf:4-robust:3 -6.44505400 -21.3370641 8.446956 0.7754247

robust:4-robust:3 3.69509380 -9.9536589 17.343846 0.9612355

dwarf:6-robust:3 -6.74870614 -20.6124130 7.115001 0.6805172

robust:6-robust:3 3.67131635 -12.6420633 19.984696 0.9825510

robust:4-dwarf:4 10.14014780 2.8445826 17.435713 0.0024999

dwarf:6-dwarf:4 -0.30365214 -7.9938531 7.386549 0.9999961

robust:6-dwarf:4 10.11637035 -1.4189310 21.651672 0.1124681

dwarf:6-robust:4 -10.44379993 -15.3075101 -5.580090 0.0000040

robust:6-robust:4 -0.02377744 -9.9020194 9.854465 1.0000000

robust:6-dwarf:6 10.42002249 0.2468429 20.593202 0.0421967

$`Treatment:Ploidy`

diff lwr upr p adj

warm:3-cold:3 NA NA NA NA

cold:4-cold:3 3.9930090 -9.794313 17.780331 0.9487366

warm:4-cold:3 8.1118526 -5.858091 22.081796 0.5033412

cold:6-cold:3 3.5186881 -10.609113 17.646489 0.9728173

warm:6-cold:3 8.7020753 -5.684973 23.089124 0.4587987

cold:4-warm:3 NA NA NA NA

warm:4-warm:3 NA NA NA NA

cold:6-warm:3 NA NA NA NA

warm:6-warm:3 NA NA NA NA

warm:4-cold:4 4.1188437 -1.396085 9.633773 0.2378740

cold:6-cold:4 -0.4743209 -6.377700 5.429058 0.9998695

warm:6-cold:4 4.7090663 -1.790340 11.208472 0.2669227

cold:6-warm:4 -4.5931646 -10.911309 1.724980 0.2636324

warm:6-warm:4 0.5902227 -6.288102 7.468547 0.9998196

warm:6-cold:6 5.1833872 -2.010137 12.376911 0.2723369

*Leaf Area Cm^2^*

*
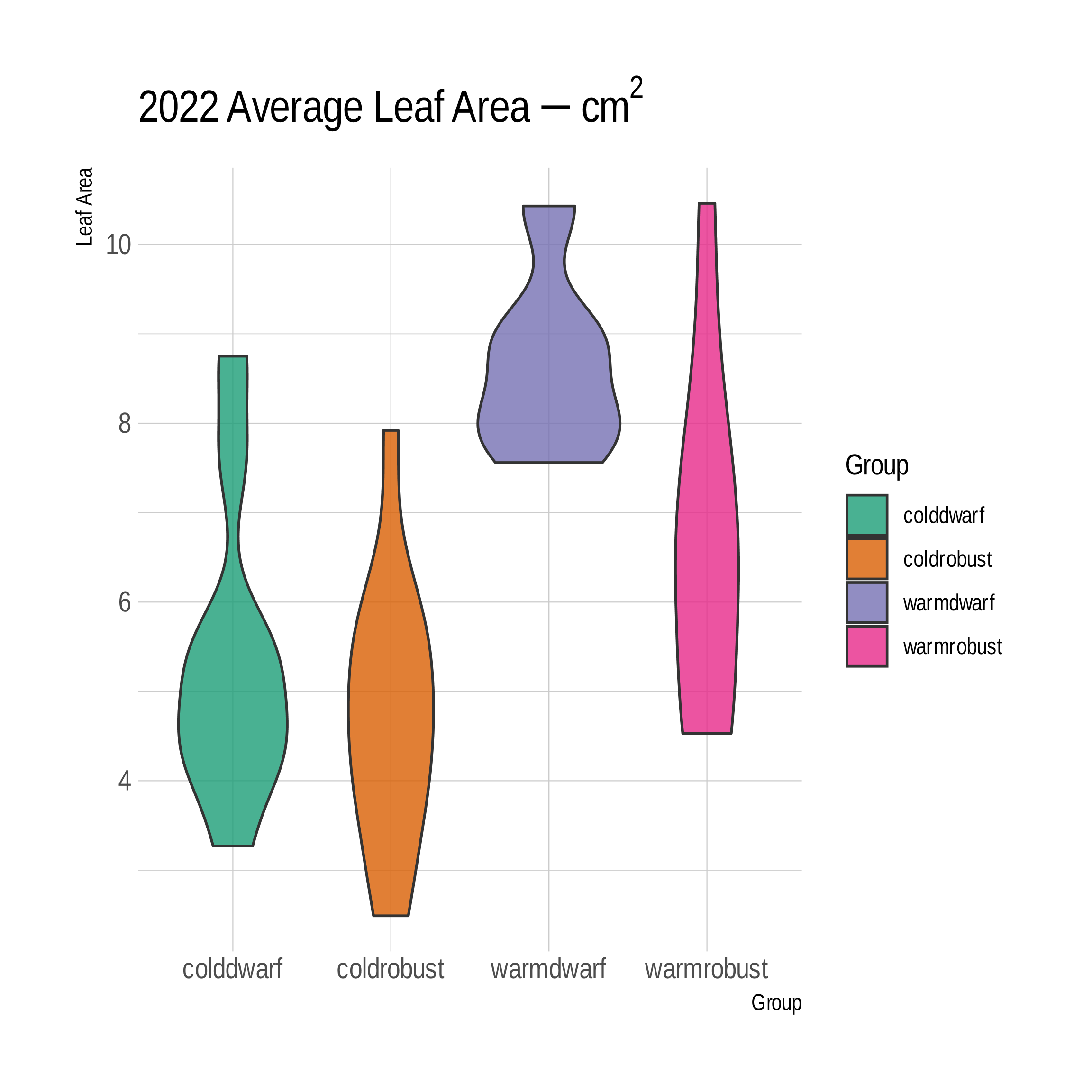
*

***Shapiro Wilk***

Shapiro-Wilk normality test

data: a_leaf_area$LA_AVG

W = 0.95534, p-value = 0.1244

***Levene***

> leveneTest(a_leaf_area$LA_AVG, a_leaf_area$Morph) #good

Levene's Test for Homogeneity of Variance (center = median)

Df F value Pr(>F)

group 1 2.0144 0.1642

37

> leveneTest(a_leaf_area$LA_AVG, a_leaf_area$Treatment) #good

Levene's Test for Homogeneity of Variance (center = median)

Df F value Pr(>F)

group 1 0.8945 0.3504

37

> leveneTest(a_leaf_area$LA_AVG, a_leaf_area$Ploidy) #good

Levene's Test for Homogeneity of Variance (center = median)

Df F value Pr(>F)

group 2 1.9084 0.163

36

***AOV***

Df Sum Sq Mean Sq F value Pr(>F)

Morph 1 9.76 9.76 3.478 0.0717 .

Treatment 1 56.91 56.91 20.275 **8.88e-05 *****

Ploidy 2 0.06 0.03 0.011 0.9890

Morph:Treatment 1 4.53 4.53 1.615 0.2132

Morph:Ploidy 1 0.59 0.59 0.209 0.6505

Treatment:Ploidy 1 0.79 0.79 0.283 0.5985

Residuals 31 87.02 2.81

---

Signif. codes: 0 ‘***’ 0.001 ‘**’ 0.01 ‘*’ 0.05 ‘.’ 0.1 ‘ ’ 1

***Tukey HSD***

> TukeyHSD(aovleafarea)

Tukey multiple comparisons of means

95% family-wise confidence level

Fit: aov(formula = LA_AVG ~ (Morph + Treatment + Ploidy)^2, data = a_leaf_area)

$Morph

diff lwr upr p adj

robust-dwarf -1.017255 -2.129656 0.09514537 **0.0716621**

$Treatment

diff lwr upr p adj

warm-cold 2.451548 1.339147 3.563949 **9.09e-05**

$Ploidy

diff lwr upr p adj

4-3 0.075381734 -4.129831 4.280594 0.9989275

6-3 0.008503335 -4.270707 4.287713 0.9999868

6-4 -0.066878399 -1.476886 1.343129 0.9925169

$`Morph:Treatment`

diff lwr upr p adj

robust:cold-dwarf:cold -0.6030311 -2.51570058 1.3096384 **0.8273214**

dwarf:warm-dwarf:cold 3.3063735 0.95818981 5.6545571 **0.0031876**

robust:warm-dwarf:cold 1.2960000 -0.73758669 3.3295867 0.3260519

dwarf:warm-robust:cold 3.9094045 1.66512374 6.1536853 **0.0002618**

robust:warm-robust:cold 1.8990311 -0.01363844 3.8117006 0.0522090

robust:warm-dwarf:warm -2.0103735 -4.35855711 0.3378102 0.1144501

$`Morph:Ploidy`

diff lwr upr p adj

robust:3-dwarf:3 NA NA NA NA

dwarf:4-dwarf:3 NA NA NA NA

robust:4-dwarf:3 NA NA NA NA

dwarf:6-dwarf:3 NA NA NA NA

robust:6-dwarf:3 NA NA NA NA

dwarf:4-robust:3 0.9743751 -4.596199 6.544950 0.9944537

robust:4-robust:3 0.1049472 -5.105848 5.315742 0.9999999

dwarf:6-robust:3 1.1111553 -4.200179 6.422490 0.9874079

robust:6-robust:3 -0.4611777 -6.689269 5.766914 0.9999127

robust:4-dwarf:4 -0.8694279 -3.412036 1.673180 0.9013716

dwarf:6-dwarf:4 0.1367802 -2.605981 2.879541 0.9999877

robust:6-dwarf:4 -1.4355528 -5.690149 2.819044 0.9062341

dwarf:6-robust:4 1.0062081 -0.902673 2.915089 0.6047349

robust:6-robust:4 -0.5661250 -4.337422 3.205172 0.9972949

robust:6-dwarf:6 -1.5723330 -5.481369 2.336703 0.8234048

$`Treatment:Ploidy`

diff lwr upr p adj

warm:3-cold:3 NA NA NA NA

cold:4-cold:3 0.03731389 -5.2263842 5.30101193 1.0000000

warm:4-cold:3 2.65102986 -2.6603044 7.96236415 0.6576014

cold:6-cold:3 0.26563141 -5.1280541 5.65931688 0.9999884

warm:6-cold:3 2.38102780 -3.1895466 7.95160220 0.7841889

cold:4-warm:3 NA NA NA NA

warm:4-warm:3 NA NA NA NA

cold:6-warm:3 NA NA NA NA

warm:6-warm:3 NA NA NA NA

warm:4-cold:4 2.61371597 0.5648243 4.66260765 0.0063096

cold:6-cold:4 0.22831752 -2.0254633 2.48209837 0.9995933

warm:6-cold:4 2.34371391 -0.3056227 4.99305049 0.1072231

cold:6-warm:4 -2.38539845 -4.7482956 -0.02250129 0.0467951

warm:6-warm:4 -0.27000206 -3.0127633 2.47275917 0.9996464

warm:6-cold:6 2.11539639 -0.7836224 5.01441522 0.2600077

*
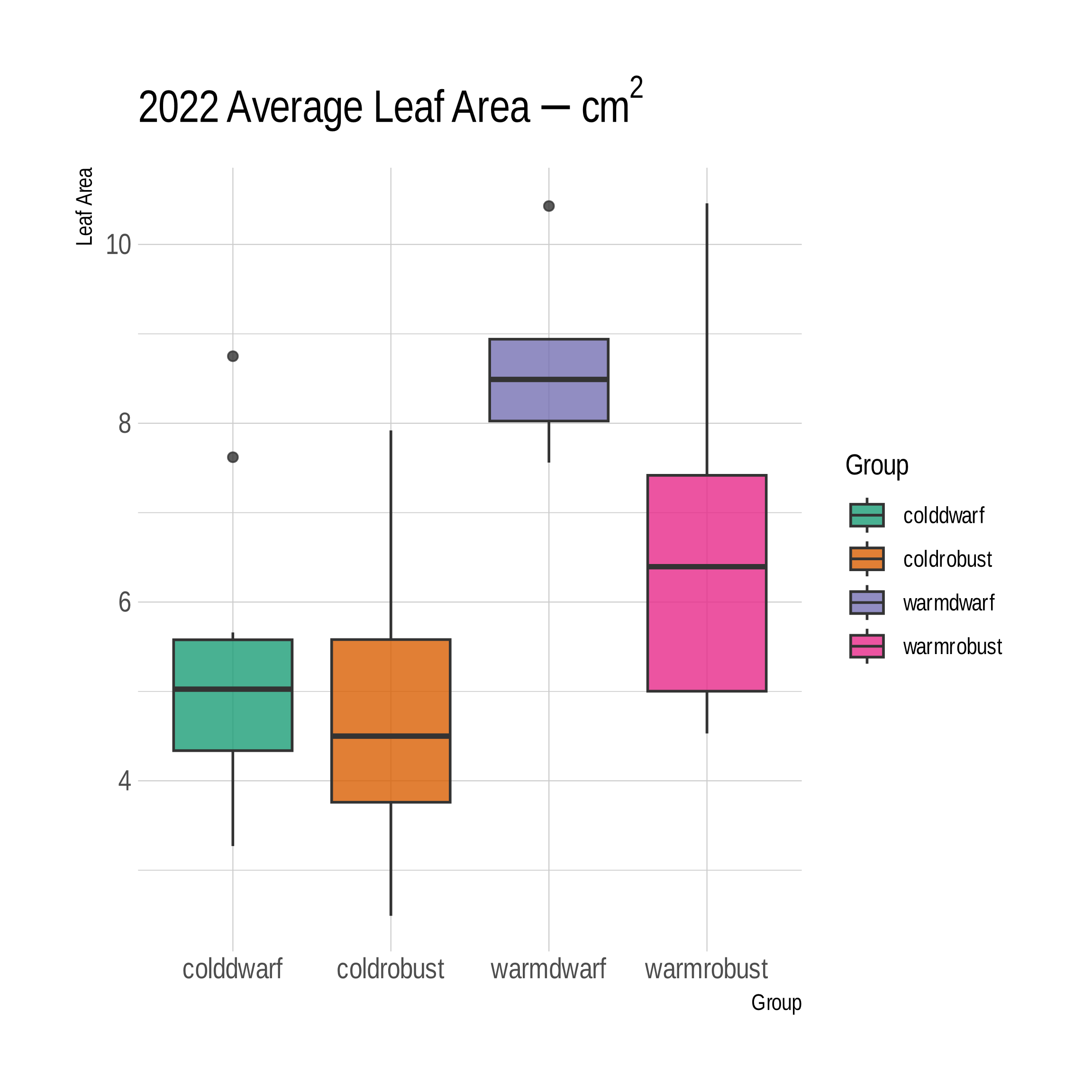
*

*Basal Leaf Number per Plant*

*
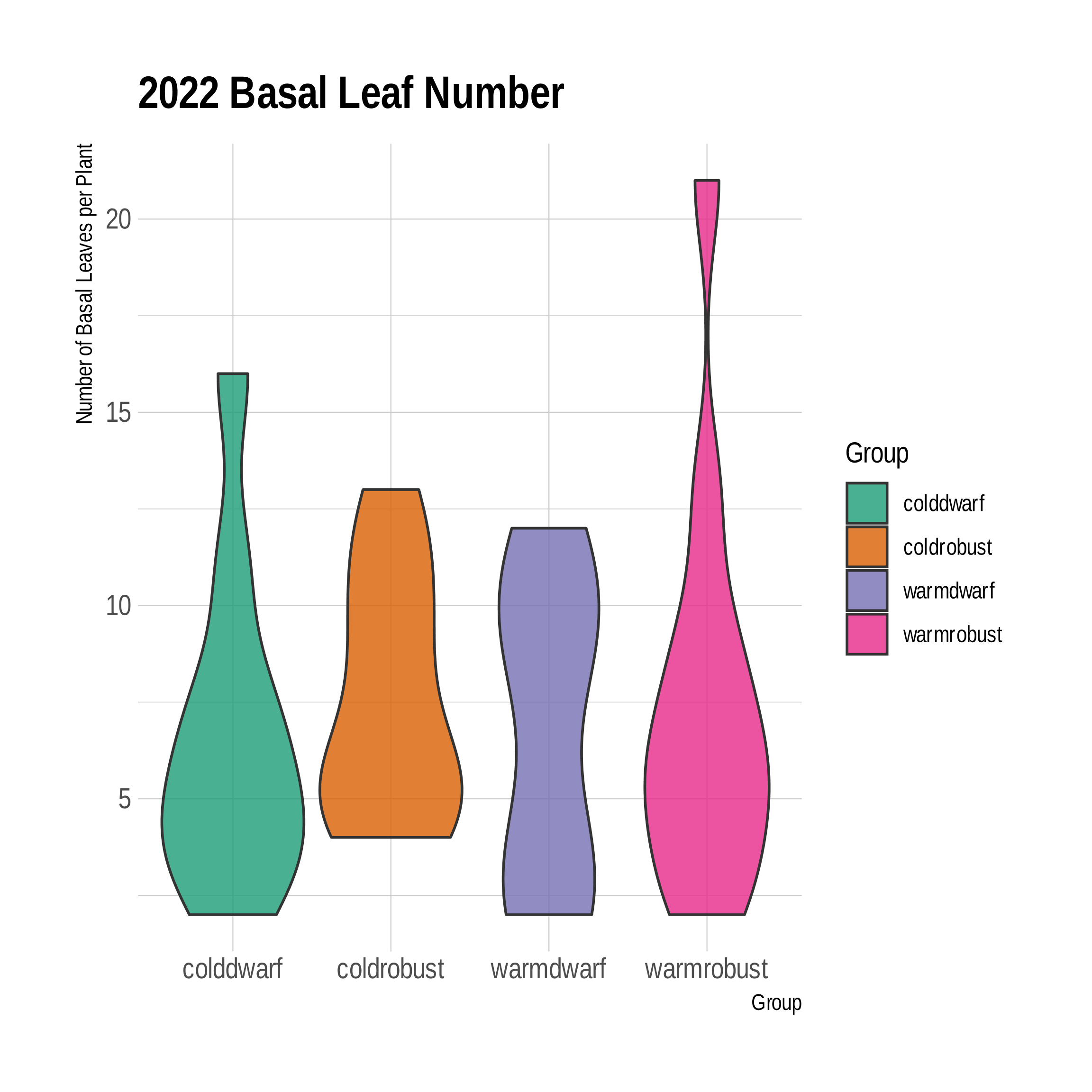
*

***Shapiro Wilk***

Shapiro-Wilk normality test

data: a_nBl$n_BL

W = 0.94157, p-value = **0.001626**

***Levene***

> leveneTest(a_leaves$n_BL, a_leaves$Morph) #

Levene's Test for Homogeneity of Variance (center = median)

Df F value Pr(>F)

group 1 0.1017 0.7514

42

> leveneTest(a_leaves$n_BL, a_leaves$Treatment) #

Levene's Test for Homogeneity of Variance (center = median)

Df F value Pr(>F)

group 1 0.8614 0.3587

42

> leveneTest(a_leaves$n_BL, a_leaves$Ploidy) #

Levene's Test for Homogeneity of Variance (center = median)

Df F value Pr(>F)

group 2 0.622 0.5418

41

***AOV***

> aovleave <- aov(n_BL ~ (Morph + Treatment + Ploidy)^2, data = a_leaves)

> summary(aovleave)

Df Sum Sq Mean Sq F value Pr(>F)

Morph 1 7.5 7.541 0.400 0.531

Treatment 1 0.0 0.033 0.002 0.967

Ploidy 2 8.6 4.285 0.227 0.798

Morph:Treatment 1 0.3 0.255 0.014 0.908

Morph:Ploidy 1 22.2 22.193 1.178 0.285

Treatment:Ploidy 1 3.1 3.078 0.163 0.688

Residuals 36 678.2 18.839

***Tukey HSD***

> TukeyHSD(aovleave)

Tukey multiple comparisons of means

95% family-wise confidence level

Fit: aov(formula = n_BL ~ (Morph + Treatment + Ploidy)^2, data = a_leaves)

$Morph

diff lwr upr p adj

robust-dwarf 0.8357895 -1.843382 3.51496 0.5309406

$Treatment

diff lwr upr p adj

warm-cold 0.05485017 -2.60204 2.711741 0.9668345

$Ploidy

diff lwr upr p adj

4-3 1.7587402 -9.038352 12.555832 0.9165328

6-3 1.0984529 -9.858787 12.055693 0.9674717

6-4 -0.6602873 -4.054948 2.734374 0.8832650

$`Morph:Treatment`

diff lwr upr p adj

robust:cold-dwarf:cold 0.98080923 -3.936166 5.897785 0.9493517

dwarf:warm-dwarf:cold 0.22830860 -5.142768 5.599385 0.9994529

robust:warm-dwarf:cold 0.90398928 -4.101269 5.909248 0.9616271

dwarf:warm-robust:cold -0.75250063 -5.821523 4.316522 0.9780312

robust:warm-robust:cold -0.07681996 -4.756471 4.602832 0.9999683

robust:warm-dwarf:warm 0.67568067 -4.479021 5.830383 0.9846614

$`Morph:Ploidy`

diff lwr upr p adj

robust:3-dwarf:3 NA NA NA NA

dwarf:4-dwarf:3 NA NA NA NA

robust:4-dwarf:3 NA NA NA NA

dwarf:6-dwarf:3 NA NA NA NA

robust:6-dwarf:3 NA NA NA NA

dwarf:4-robust:3 0.4708170 -13.633986 14.575620 0.9999984

robust:4-robust:3 1.8820494 -11.469947 15.234046 0.9981026

dwarf:6-robust:3 0.7442188 -12.807237 14.295675 0.9999813

robust:6-robust:3 -2.0316574 -18.025000 13.961686 0.9988481

robust:4-dwarf:4 1.4112324 -4.603076 7.425540 0.9800217

dwarf:6-dwarf:4 0.2734019 -6.171601 6.718405 0.9999948

robust:6-dwarf:4 -2.5024744 -13.164703 8.159754 0.9799996

dwarf:6-robust:4 -1.1378306 -5.706025 3.430364 0.9740699

robust:6-robust:4 -3.9137068 -13.558056 5.730642 0.8238214

robust:6-dwarf:6 -2.7758763 -12.694527 7.142774 0.9574241

$`Treatment:Ploidy`

diff lwr upr p adj

warm:3-cold:3 NA NA NA NA

cold:4-cold:3 1.6485201 -11.868322 15.165362 0.9990537

warm:4-cold:3 1.9239611 -11.592881 15.440803 0.9980121

cold:6-cold:3 1.4098266 -12.440815 15.260468 0.9996069

warm:6-cold:3 0.7980929 -13.162041 14.758227 0.9999771

cold:4-warm:3 NA NA NA NA

warm:4-warm:3 NA NA NA NA

cold:6-warm:3 NA NA NA NA

warm:6-warm:3 NA NA NA NA

warm:4-cold:4 0.2754409 -4.660212 5.211094 0.9999797

cold:6-cold:4 -0.2386936 -6.026259 5.548872 0.9999955

warm:6-cold:4 -0.8504272 -6.895343 5.194488 0.9981200

cold:6-warm:4 -0.5141345 -6.301700 5.273431 0.9997982

warm:6-warm:4 -1.1258681 -7.170784 4.919047 0.9929632

warm:6-cold:6 -0.6117337 -7.370155 6.146687 0.9997787

*Flower Number per Plant*

*
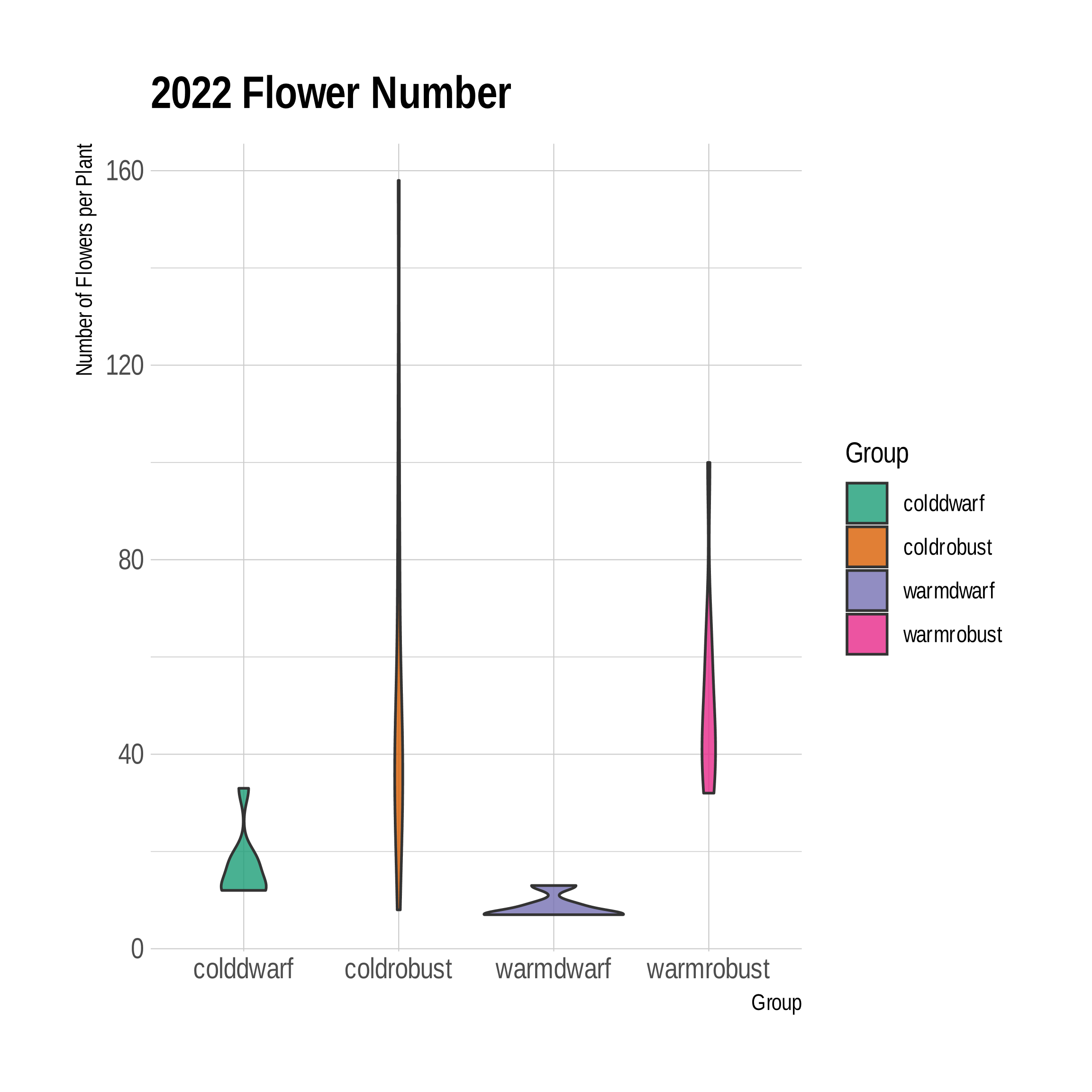
*

***Shapiro Wilk***

Shapiro-Wilk normality test

data: a_flowers$n_Fl

W = 0.82219, p-value = 4.638e-05

***Levene***

> leveneTest(a_flowers$n_Fl, a_flowers$Morph)#good

Levene's Test for Homogeneity of Variance (center = median)

Df F value Pr(>F)

group 1 5.0181 **0.03173 ***

34

---

Signif. codes: 0 ‘***’ 0.001 ‘**’ 0.01 ‘*’ 0.05 ‘.’ 0.1 ‘ ’ 1

> leveneTest(a_flowers$n_Fl, a_flowers$Treatment)#good

Levene's Test for Homogeneity of Variance (center = median)

Df F value Pr(>F)

group 1 0.2176 0.6438

34

> leveneTest(a_flowers$n_Fl, a_flowers$Ploidy)#good

Levene's Test for Homogeneity of Variance (center = median)

Df F value Pr(>F)

group 2 2.0437 0.1456

33

***AOV***

> aovflowers <- aov(n_Fl ~ (Morph + Treatment + Ploidy)^2, data = a_flowers)

> summary(aovflowers)

Df Sum Sq Mean Sq F value Pr(>F)

Morph 1 13371 13371 16.230 **0.000389 *****

Treatment 1 315 315 0.383 0.541108

Ploidy 2 1043 522 0.633 0.538294

Morph:Treatment 1 18 18 0.022 0.882783

Morph:Ploidy 1 81 81 0.099 0.755751

Treatment:Ploidy 1 561 561 0.682 0.416047

Residuals 28 23068 824

---

Signif. codes: 0 ‘***’ 0.001 ‘**’ 0.01 ‘*’ 0.05 ‘.’ 0.1 ‘ ’ 1

***Tukey HSD***

> TukeyHSD(aovflowers)

Tukey multiple comparisons of means

95% family-wise confidence level

Fit: aov(formula = n_Fl ~ (Morph + Treatment + Ploidy)^2, data = a_flowers)

$Morph

diff lwr upr p adj

robust-dwarf 40.12375 19.72256 60.52493 **0.0003891**

$Treatment

diff lwr upr p adj

warm-cold -5.996369 -25.87268 13.87994 0.541588

$Ploidy

diff lwr upr p adj

4-3 20.407496 -52.14066 92.95565 0.7677468

6-3 12.943103 -60.97754 86.86374 0.9020715

6-4 -7.464393 -32.75528 17.82650 0.7477787

$`Morph:Treatment`

diff lwr upr p adj

robust:cold-dwarf:cold 41.595451 6.380372 76.810530 **0.0159202**

dwarf:warm-dwarf:cold -4.127322 -48.803640 40.548996 0.9942370

robust:warm-dwarf:cold 34.559437 -2.613507 71.732380 0.0755137

dwarf:warm-robust:cold -45.722773 -86.962451 -4.483095 **0.0254818**

robust:warm-robust:cold -7.036014 -39.999106 25.927077 0.9364366

robust:warm-dwarf:warm 38.686759 -4.236859 81.610377 0.0886677

$`Morph:Ploidy`

diff lwr upr p adj

robust:3-dwarf:3 NA NA NA NA

dwarf:4-dwarf:3 NA NA NA NA

robust:4-dwarf:3 NA NA NA NA

dwarf:6-dwarf:3 NA NA NA NA

robust:6-dwarf:3 NA NA NA NA

dwarf:4-robust:3 -15.658970 -116.94013 85.62219 0.9967564

robust:4-robust:3 19.798904 -70.07921 109.67702 0.9835140

dwarf:6-robust:3 -24.904941 -116.89812 67.08824 0.9598627

robust:6-robust:3 1.564597 -105.86030 108.98949 1.0000000

robust:4-dwarf:4 35.457874 -18.84810 89.76385 0.3699943

dwarf:6-dwarf:4 -9.245972 -66.98512 48.49317 0.9961737

robust:6-dwarf:4 17.223567 -62.84622 97.29335 0.9851708

dwarf:6-robust:4 -44.703845 -78.67458 -10.73311 0.0048156

robust:6-robust:4 -18.234307 -83.28331 46.81470 0.9536246

robust:6-dwarf:6 26.469538 -41.47193 94.41101 0.8375747

$`Treatment:Ploidy`

diff lwr upr p adj

warm:3-cold:3 NA NA NA NA

cold:4-cold:3 21.7823281 -69.00828 112.57293 0.9760150

warm:4-cold:3 12.1576271 -80.29900 104.61425 0.9984984

cold:6-cold:3 9.5062288 -85.23362 104.24608 0.9995943

warm:6-cold:3 9.8666809 -84.87317 104.60653 0.9995136

cold:4-warm:3 NA NA NA NA

warm:4-warm:3 NA NA NA NA

cold:6-warm:3 NA NA NA NA

warm:6-warm:3 NA NA NA NA

warm:4-cold:4 -9.6247010 -47.09938 27.84998 0.9678559

cold:6-cold:4 -12.2760993 -55.07520 30.52300 0.9490396

warm:6-cold:4 -11.9156472 -54.71475 30.88345 0.9549136

cold:6-warm:4 -2.6513983 -48.87971 43.57692 0.9999742

warm:6-warm:4 -2.2909462 -48.51926 43.93737 0.9999875

warm:6-cold:6 0.3604521 -50.28013 51.00103 1.0000000

*Number of Stems per Plant*

*
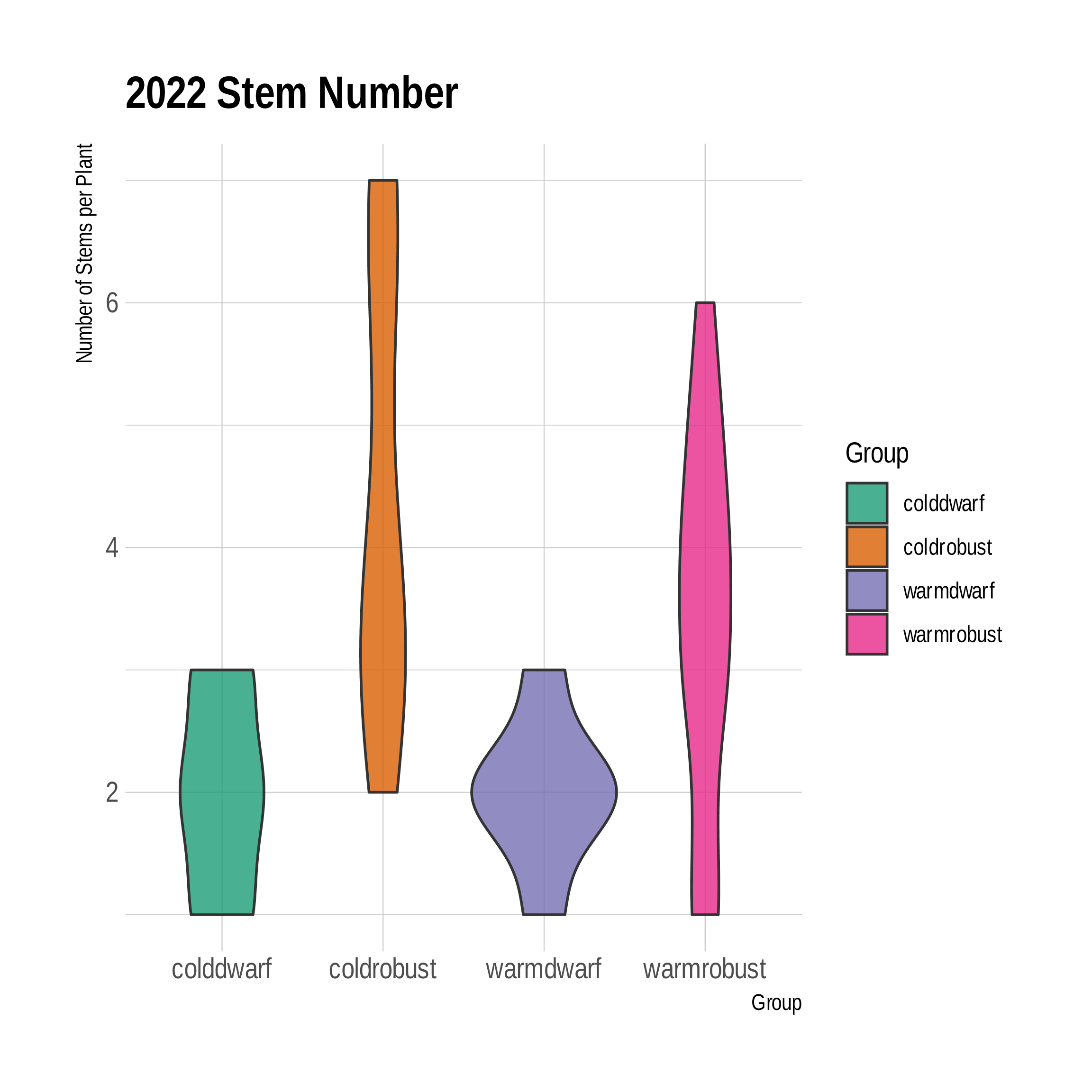
*

***Shapiro Wilk***

Shapiro-Wilk normality test

data: a_stems$n_St

W = 0.88871, p-value = **0.0007826**

***Levene***

> leveneTest(a_stems$n_St, a_stems$Morph) #good

Levene's Test for Homogeneity of Variance (center = median)

Df F value Pr(>F)

group 1 10.273 **0.002692 ****

39

---

Signif. codes: 0 ‘***’ 0.001 ‘**’ 0.01 ‘*’ 0.05 ‘.’ 0.1 ‘ ’ 1

> leveneTest(a_stems$n_St, a_stems$Treatment) #good

Levene's Test for Homogeneity of Variance (center = median)

Df F value Pr(>F)

group 1 0.232 0.6328

39

> leveneTest(a_stems$n_St, a_stems$Ploidy) #good

Levene's Test for Homogeneity of Variance (center = median)

Df F value Pr(>F)

group 2 3.7759 **0.03194 ***

38

---

Signif. codes: 0 ‘***’ 0.001 ‘**’ 0.01 ‘*’ 0.05 ‘.’ 0.1 ‘ ’ 1

***AOV***

> aovstems <- aov(n_St ~ (Morph+Treatment+Ploidy)^2, data = a_stems)

> summary(aovstems)

Df Sum Sq Mean Sq F value Pr(>F)

Morph 1 37.48 37.48 17.699 **0.000186 *****

Treatment 1 4.26 4.26 2.010 0.165684

Ploidy 2 1.76 0.88 0.415 0.663799

Morph:Treatment 1 3.11 3.11 1.471 0.233847

Morph:Ploidy 1 0.89 0.89 0.419 0.522119

Treatment:Ploidy 1 3.07 3.07 1.449 0.237220

Residuals 33 69.88 2.12

---

Signif. codes: 0 ‘***’ 0.001 ‘**’ 0.01 ‘*’ 0.05 ‘.’ 0.1 ‘ ’ 1

***Tukey HSD***

> TukeyHSD(aovstems)

Tukey multiple comparisons of means

95% family-wise confidence level

Fit: aov(formula = n_St ~ (Morph + Treatment + Ploidy)^2, data = a_stems)

$Morph

diff lwr upr p adj

robust-dwarf 1.96 1.012156 2.907844 **0.0001862**

$Treatment

diff lwr upr p adj

warm-cold -0.6457005 -1.57738 0.2859794 0.1678899

$Ploidy

diff lwr upr p adj

4-3 1.305613306 -2.333097 4.944323 0.6562276

6-3 1.304161304 -2.391855 5.000177 0.6652650

6-4 -0.001452001 -1.185124 1.182220 0.9999950

$`Morph:Treatment`

diff lwr upr p adj

robust:cold-dwarf:cold 2.50079520 0.8451582 4.1564321 **0.0014372**

dwarf:warm-dwarf:cold 0.05916957 -1.9734553 2.0917944 0.9998212

robust:warm-dwarf:cold 1.42036476 -0.2649987 3.1057283 0.1235463

dwarf:warm-robust:cold -2.44162563 -4.3843105 -0.4989407 **0.0091781**

robust:warm-robust:cold -1.08043043 -2.6561560 0.4952951 0.2668849

robust:warm-dwarf:warm 1.36119520 -0.6068854 3.3292758 0.2599152

$`Morph:Ploidy`

diff lwr upr p adj

robust:3-dwarf:3 NA NA NA NA

dwarf:4-dwarf:3 NA NA NA NA

robust:4-dwarf:3 NA NA NA NA

dwarf:6-dwarf:3 NA NA NA NA

robust:6-dwarf:3 NA NA NA NA

dwarf:4-robust:3 -0.9840861 -5.90316077 3.9349886 0.9899427

robust:4-robust:3 1.3655586 -3.13307873 5.8641960 0.9391222

dwarf:6-robust:3 -0.5876069 -5.16701625 3.9918024 0.9987521

robust:6-robust:3 0.8947708 -4.49380556 6.2833471 0.9957497

robust:4-dwarf:4 2.3496447 -0.04187378 4.7411633 0.0564650

dwarf:6-dwarf:4 0.3964791 -2.14372010 2.9366784 0.9968237

robust:6-dwarf:4 1.8788569 -1.93144201 5.6891557 0.6722115

dwarf:6-robust:4 -1.9531656 -3.53210660 -0.3742246 0.0083921

robust:6-robust:4 -0.4707879 -3.72022167 2.7786459 0.9977684

robust:6-dwarf:6 1.4823777 -1.87799003 4.8427455 0.7644394

$`Treatment:Ploidy`

diff lwr upr p adj

warm:3-cold:3 NA NA NA NA

cold:4-cold:3 1.4446126 -3.109566 5.9987908 0.9274610

warm:4-cold:3 0.4679520 -4.111457 5.0473613 0.9995862

cold:6-cold:3 1.0469280 -3.619716 5.7135720 0.9831598

warm:6-cold:3 0.8574293 -3.894848 5.6097069 0.9937416

cold:4-warm:3 NA NA NA NA

warm:4-warm:3 NA NA NA NA

cold:6-warm:3 NA NA NA NA

warm:6-warm:3 NA NA NA NA

warm:4-cold:4 -0.9766605 -2.707515 0.7541935 0.5376328

cold:6-cold:4 -0.3976845 -2.347667 1.5522981 0.9890237

warm:6-cold:4 -0.5871833 -2.734043 1.5596769 0.9603296

cold:6-warm:4 0.5789760 -1.429228 2.5871798 0.9506302

warm:6-warm:4 0.3894773 -1.810400 2.5893543 0.9942672

warm:6-cold:6 -0.1894987 -2.565638 2.1866401 0.9998773

**2023**

Outliers were identified and removed from natural log transformed group (cold dwarf, robust warm, etc.) datasets using the Grubbs Test (implemented in Rstudio using the grubbs.test function in the outliers package). Both single and double outliers on either end were tested for, as well as opposite outliers.

*Plant Height*

*
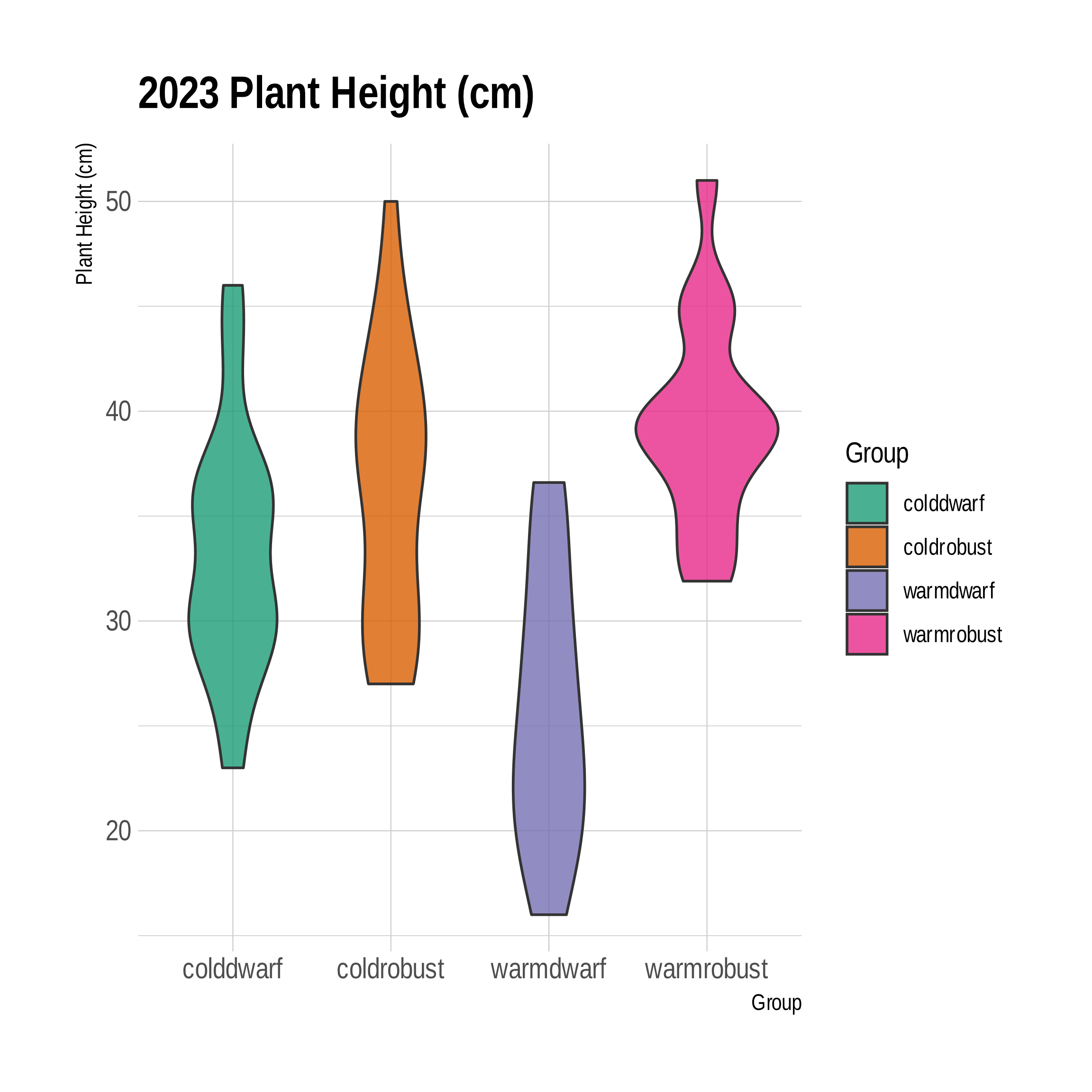
*

***Shapiro Wilk***

Shapiro-Wilk normality test

data: a_height$HEIGHT_L

W = 0.98728, p-value = 0.6856

***Levene***

> leveneTest(a_height$HEIGHT_L, a_height$Morph) #

Levene's Test for Homogeneity of Variance (center = median)

Df F value Pr(>F)

group 1 1.3743 0.245

70

> leveneTest(a_height$HEIGHT_L, a_height$Treatment) #

Levene's Test for Homogeneity of Variance (center = median)

Df F value Pr(>F)

group 1 4.1938 0.04433 *

70

---

Signif. codes: 0 ‘***’ 0.001 ‘**’ 0.01 ‘*’ 0.05 ‘.’ 0.1 ‘ ’ 1

> leveneTest(a_height$HEIGHT_L, a_height$Ploidy) #

Levene's Test for Homogeneity of Variance (center = median)

Df F value Pr(>F)

group 1 1.6895 0.1979

70

***AOV***

Df Sum Sq Mean Sq F value Pr(>F)

Morph 1 1217.9 1217.9 33.879 1.97e-07 ***

Treatment 1 66.3 66.3 1.845 0.179068

Ploidy 1 15.2 15.2 0.424 0.517315

Morph:Treatment 1 511.7 511.7 14.235 0.000351 ***

Morph:Ploidy 1 13.3 13.3 0.369 0.545877

Treatment:Ploidy 1 77.6 77.6 2.160 0.146505

Residuals 65 2336.6 35.9

---

Signif. codes: 0 ‘***’ 0.001 ‘**’ 0.01 ‘*’ 0.05 ‘.’ 0.1 ‘ ’ 1

***Tukey HSD***

Tukey multiple comparisons of means

95% family-wise confidence level

Fit: aov(formula = HEIGHT_L ~ (Morph + Treatment + Ploidy)^2, data = a_height)

$Morph

diff lwr upr p adj

robust-dwarf 8.238235 5.411552 11.06492 **2e-07**

$Treatment

diff lwr upr p adj

warm-cold -1.919814 -4.746498 0.9068692 0.1796625

$Ploidy

diff lwr upr p adj

6-4 0.95625 -2.438083 4.350583 0.5756204

$`Morph:Treatment`

diff lwr upr p adj

robust:cold-dwarf:cold 3.050420 -2.1073548 8.208195 0.4087565

dwarf:warm-dwarf:cold -7.547059 -12.9695048 -2.124613 **0.0027058**

robust:warm-dwarf:cold 6.211765 0.7893187 11.634211 **0.0184522**

dwarf:warm-robust:cold -10.597479 -15.7552539 -5.439704 **0.0000056**

robust:warm-robust:cold 3.161345 -1.9964304 8.319119 0.3768494

robust:warm-dwarf:warm 13.758824 8.3363775 19.181270 **0.0000000**

$`Morph:Ploidy`

diff lwr upr p adj

robust:4-dwarf:4 8.5018701 4.039958 12.963783 **0.0000246**

dwarf:6-dwarf:4 0.8764912 -4.583874 6.336857 0.9743078

robust:6-dwarf:4 13.1778638 -3.041839 29.397567 0.1507664

dwarf:6-robust:4 -7.6253789 -12.464426 -2.786331 **0.0005506**

robust:6-robust:4 4.6759936 -11.345228 20.697216 0.8678677

robust:6-dwarf:6 12.3013725 -4.026104 28.628849 0.2036223

$`Treatment:Ploidy`

diff lwr upr p adj

warm:4-cold:4 -1.0964717 -5.346070 3.153126 0.9042048

cold:6-cold:4 3.2212907 -3.394270 9.836851 0.5763802

warm:6-cold:4 -1.9651598 -7.951103 4.020783 0.8224855

cold:6-warm:4 4.3177625 -2.442455 11.077980 0.3402941

warm:6-warm:4 -0.8686881 -7.014127 5.276751 0.9821802

warm:6-cold:6 -5.1864506 -13.153443 2.780542 0.3235628

*Leaf Area (mm^2^)*

*
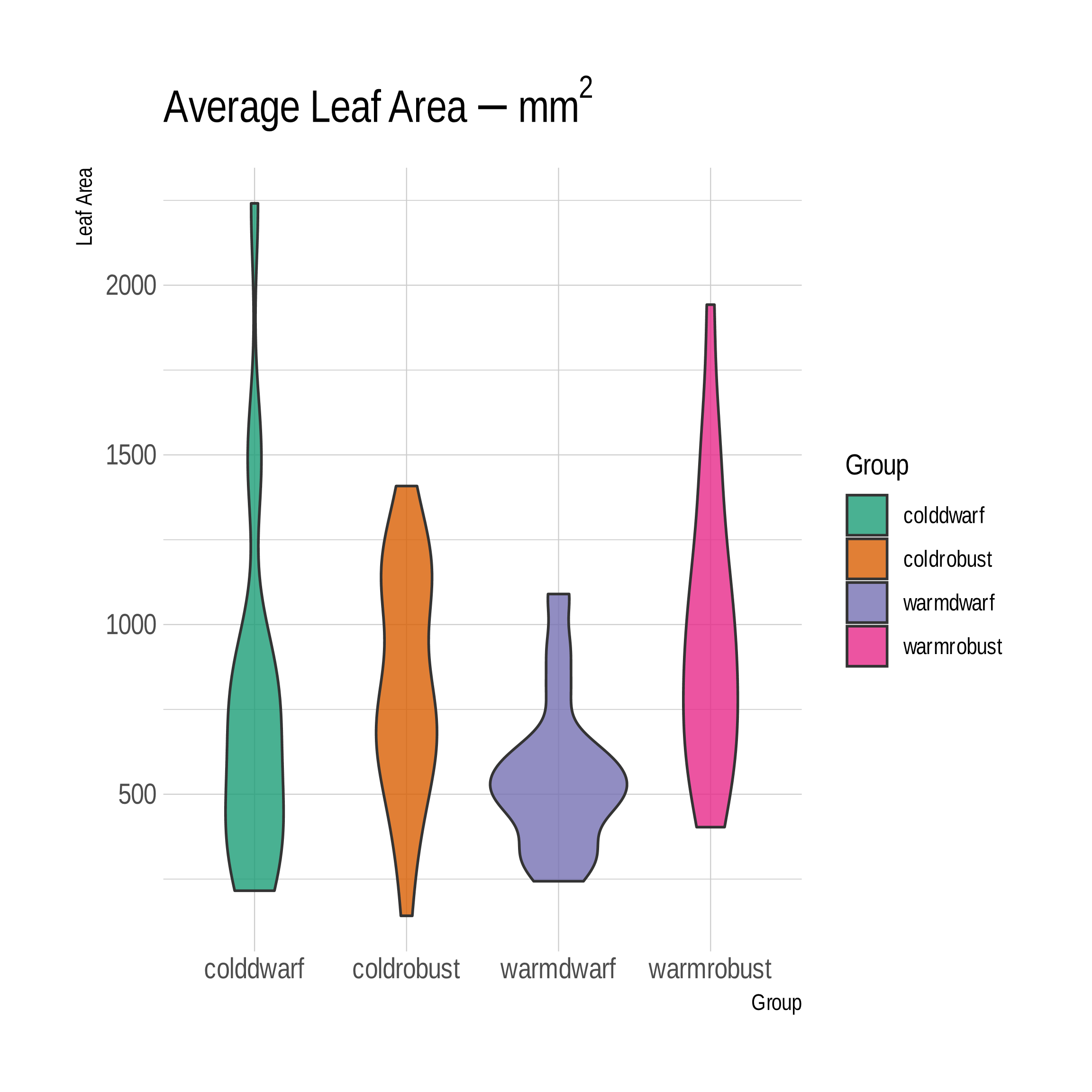
*

***Shapiro Wilk***

Shapiro-Wilk normality test

data: a_leaf_area$LA_AVG

W = 0.92465, p-value = **0.0002339**

***Levene***

> leveneTest(a_leaf_area$LA_AVG, a_leaf_area$Morph)

Levene's Test for Homogeneity of Variance (center = median)

Df F value Pr(>F)

group 1 0.0684 0.7943

74

> leveneTest(a_leaf_area$LA_AVG, a_leaf_area$Treatment)

Levene's Test for Homogeneity of Variance (center = median)

Df F value Pr(>F)

group 1 0.0506 0.8227

74

> leveneTest(a_leaf_area$LA_AVG, a_leaf_area$Ploidy)

Levene's Test for Homogeneity of Variance (center = median)

Df F value Pr(>F)

group 1 0.2744 0.602

74

***AOV***

Df Sum Sq Mean Sq F value Pr(>F)

Morph 1 1368450 1368450 10.582 0.00177 **

Treatment 1 35780 35780 0.277 0.60057

Ploidy 1 22270 22270 0.172 0.67944

Morph:Treatment 1 365479 365479 2.826 0.09726 .

Morph:Ploidy 1 1212857 1212857 9.379 0.00313 **

Treatment:Ploidy 1 403 403 0.003 0.95562

Residuals 69 8922824 129316

---

Signif. Codes: 0 ‘***’ 0.001 ‘**’ 0.01 ‘*’ 0.05 ‘.’ 0.1 ‘ ’ 1

***Tukey HSD***

Tukey multiple comparisons of means

95% family-wise confidence level

Fit: aov(formula = LA_AVG ~ (Morph + Treatment + Ploidy)^2, data = a_leaf_area)

$Morph

diff lwr upr p adj

robust-dwarf 268.7448 103.9349 433.5546 **0.0017691**

$Treatment

diff lwr upr p adj

warm-cold -43.45533 -208.2652 121.3545 0.6005705

$Ploidy

diff lwr upr p adj

6-4 -35.42416 -232.9 162.0517 0.7215384

$`Morph:Treatment`

diff lwr upr p adj

robust:cold-dwarf:cold 137.14576 -162.61990 436.91143 0.6261042

dwarf:warm-dwarf:cold -189.89702 -505.97070 126.17666 0.3956528

robust:warm-dwarf:cold 225.40983 -81.75857 532.57823 0.2243682

dwarf:warm-robust:cold -327.04278 -635.92722 -18.15834 **0.0338439**

robust:warm-robust:cold 88.26406 -211.50160 388.02973 0.8653991

robust:warm-dwarf:warm 415.30685 99.23317 731.38052 0.0050493

$`Morph:Ploidy`

diff lwr upr p adj

robust:4-dwarf:4 171.7170 -88.66861 432.10269 0.3131128

dwarf:6-dwarf:4 -162.1368 -479.68864 155.41499 0.5383248

robust:6-dwarf:4 1170.3946 200.25773 2140.53138 **0.0117024**

dwarf:6-robust:4 -333.8539 -614.93221 -52.77551 **0.0134656**

robust:6-robust:4 998.6775 39.85984 1957.49520 0.0380180

robust:6-dwarf:6 1332.5314 356.63702 2308.42574 0.0033156

$`Treatment:Ploidy`

diff lwr upr p adj

warm:4-cold:4 -39.185155 -286.5896 208.2193 0.9753931

cold:6-cold:4 -29.929698 -404.1681 344.3087 0.9966691

warm:6-cold:4 -80.209630 -437.4282 277.0089 0.9344298

cold:6-warm:4 9.255457 -371.8503 390.3612 0.9999053

warm:6-warm:4 -41.024475 -405.4313 323.3823 0.9908648

warm:6-cold:6 -50.279932 -510.3209 409.7611 0.9916248

*Basal Leaf Number*

*
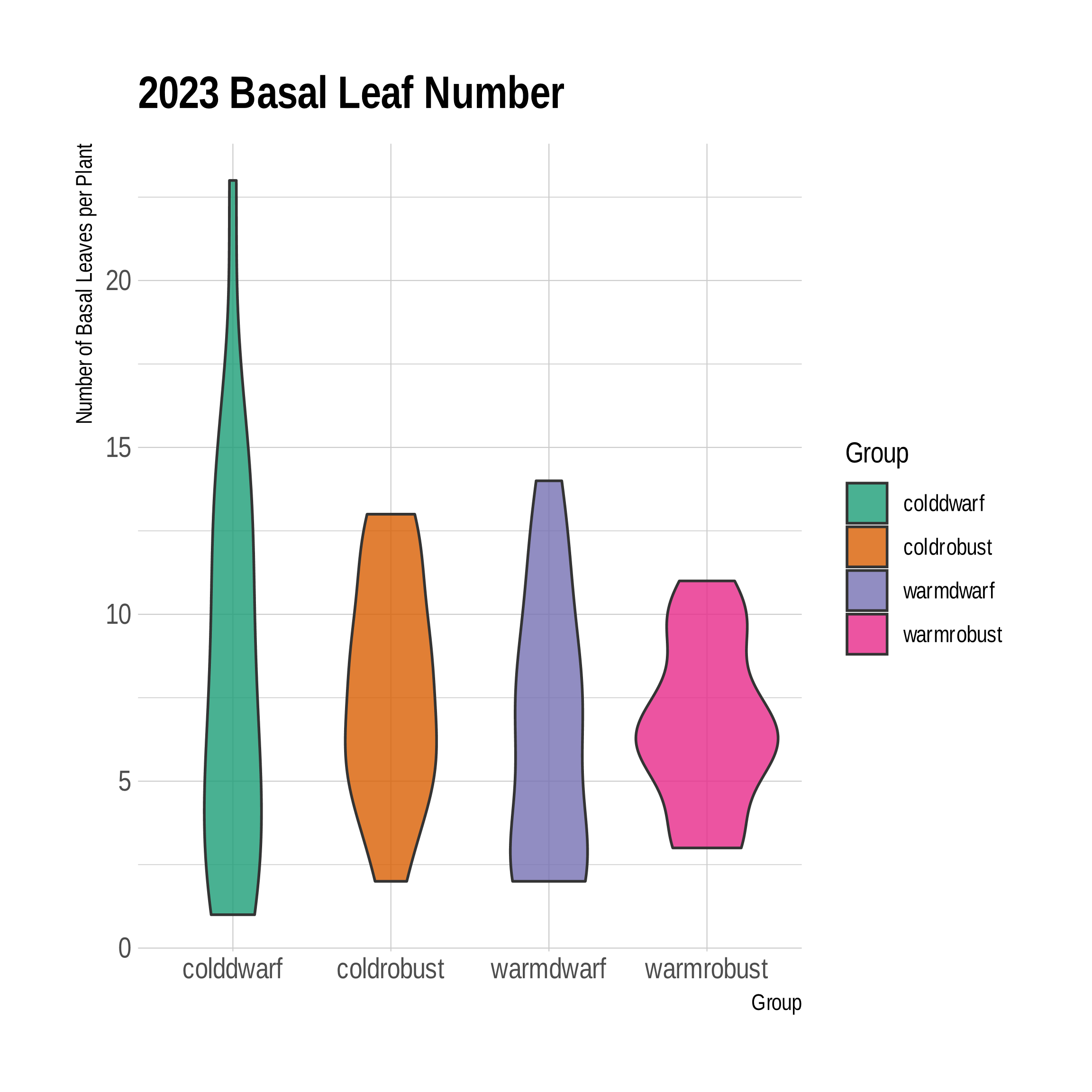
*

***Shapiro Wilk***

Shapiro-Wilk normality test

data: a_nBl$n_BL

W = 0.94157, p-value = **0.001626**

***Levene***

> leveneTest(a_leaves$n_BL, a_leaves$Morph) #

Levene's Test for Homogeneity of Variance (center = median)

Df F value Pr(>F)

group 1 9.1894 **0.003354 ****

74

---

Signif. codes: 0 ‘***’ 0.001 ‘**’ 0.01 ‘*’ 0.05 ‘.’ 0.1 ‘ ’ 1

> leveneTest(a_leaves$n_BL, a_leaves$Treatment) #

Levene's Test for Homogeneity of Variance (center = median)

Df F value Pr(>F)

group 1 3.6371 0.06039 .

74

---

Signif. codes: 0 ‘***’ 0.001 ‘**’ 0.01 ‘*’ 0.05 ‘.’ 0.1 ‘ ’ 1

> leveneTest(a_leaves$n_BL, a_leaves$Ploidy) #

Levene's Test for Homogeneity of Variance (center = median)

Df F value Pr(>F)

group 1 1.4192 0.2373

74

***AOV***

Df Sum Sq Mean Sq F value Pr(>F)

Morph 1 0.1 0.06 0.003 0.954

Treatment 1 35.5 35.53 2.043 0.157

Ploidy 1 1.0 1.04 0.060 0.808

Morph:Treatment 1 1.8 1.79 0.103 0.750

Morph:Ploidy 1 10.6 10.58 0.608 0.438

Treatment:Ploidy 1 8.1 8.08 0.465 0.498

Residuals 69 1200.1 17.39

***Tukey HSD***

Tukey multiple comparisons of means

95% family-wise confidence level

Fit: aov(formula = n_BL ~ (Morph + Treatment + Ploidy)^2, data = a_leaves)

$Morph

diff lwr upr p adj

robust-dwarf -0.05555556 -1.966932 1.855821 0.9539285

$Treatment

diff lwr upr p adj

warm-cold -1.36929 -3.280666 0.5420862 0.1574706

$Ploidy

diff lwr upr p adj

6-4 0.2416067 -2.048612 2.531826 0.8339314

$`Morph:Treatment`

diff lwr upr p adj

robust:cold-dwarf:cold -0.3428821 -3.819404 3.133639 0.9938085

dwarf:warm-dwarf:cold -1.6929689 -5.358622 1.972684 0.6189473

robust:warm-dwarf:cold -1.4210526 -4.983427 2.141322 0.7206024

dwarf:warm-robust:cold -1.3500868 -4.932363 2.232189 0.7543213

robust:warm-robust:cold -1.0781705 -4.554692 2.398351 0.8464765

robust:warm-dwarf:warm 0.2719163 -3.393737 3.937569 0.9973356

$`Morph:Ploidy`

diff lwr upr p adj

robust:4-dwarf:4 -0.14702123 -3.166834 2.872792 0.9992403

dwarf:6-dwarf:4 -0.01443773 -3.697233 3.668358 0.9999996

robust:6-dwarf:4 3.25493509 -7.996192 14.506062 0.8713473

dwarf:6-robust:4 0.13258350 -3.127212 3.392379 0.9995562

robust:6-robust:4 3.40195632 -7.717897 14.521810 0.8516496

robust:6-dwarf:6 3.26937282 -8.048527 14.587272 0.8718398

$`Treatment:Ploidy`

diff lwr upr p adj

warm:4-cold:4 -1.0956750 -3.964939 1.773589 0.7468045

cold:6-cold:4 0.8937239 -3.446492 5.233940 0.9483518

warm:6-cold:4 -1.4751564 -5.617986 2.667673 0.7848396

cold:6-warm:4 1.9893989 -2.430461 6.409259 0.6382948

warm:6-warm:4 -0.3794815 -4.605676 3.846713 0.9953068

warm:6-cold:6 -2.3688804 -7.704189 2.966429 0.6483068

*Number of Flowers per Plant*

*
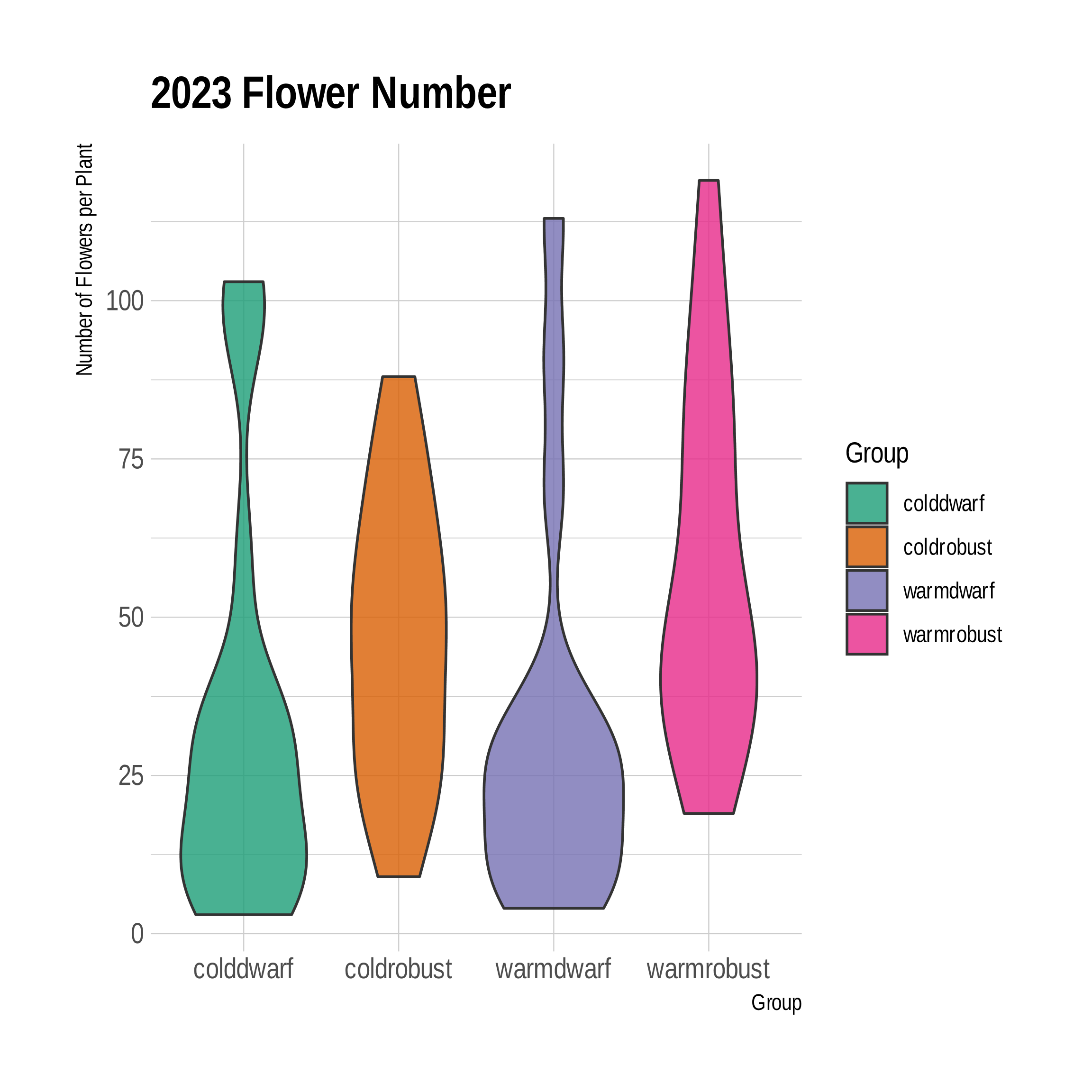
*

***Shapiro Wilk***

Shapiro-Wilk normality test

data: a_flowers$n_Fl

W = 0.92413, p-value = **0.0002211**

***Levene***

> leveneTest(a_flowers$n_Fl, a_flowers$Morph)

Levene's Test for Homogeneity of Variance (center = median)

Df F value Pr(>F)

group 1 0.0417 0.8388

74

> leveneTest(a_flowers$n_Fl, a_flowers$Treatment)

Levene's Test for Homogeneity of Variance (center = median)

Df F value Pr(>F)

group 1 0.2935 0.5896

74

> leveneTest(a_flowers$n_Fl, a_flowers$Ploidy)

Levene's Test for Homogeneity of Variance (center = median)

Df F value Pr(>F)

group 1 2.6765 0.1061

74

***AOV***

Df Sum Sq Mean Sq F value Pr(>F)

Morph 1 6675 6675 8.785 **0.00417** **

Treatment 1 532 532 0.700 0.40569

Ploidy 1 1614 1614 2.124 0.14955

Morph:Treatment 1 831 831 1.094 0.29919

Morph:Ploidy 1 4105 4105 5.402 **0.02306** *

Treatment:Ploidy 1 426 426 0.561 0.45642

Residuals 69 52426 760

---

Signif. codes: 0 ‘***’ 0.001 ‘**’ 0.01 ‘*’ 0.05 ‘.’ 0.1 ‘ ’ 1

***Tukey HSD***

Tukey multiple comparisons of means

95% family-wise confidence level

Fit: aov(formula = n_Fl ~ (Morph + Treatment + Ploidy)^2, data = a_flowers)

$Morph

diff lwr upr p adj

robust-dwarf 18.76944 6.13649 31.4024 **0.0041655**

$Treatment

diff lwr upr p adj

warm-cold 5.297863 -7.335092 17.93082 0.4056959

$Ploidy

diff lwr upr p adj

6-4 -9.535985 -24.67284 5.600872 0.2130696

$`Morph:Treatment`

diff lwr upr p adj

robust:cold-dwarf:cold 12.472429 -10.5051167 35.449974 0.4858025

dwarf:warm-dwarf:cold -1.686535 -25.9141173 22.541047 0.9977942

robust:warm-dwarf:cold 24.052632 0.5076541 47.597609 **0.0434305**

dwarf:warm-robust:cold -14.158964 -37.8354786 9.517551 0.3998072

robust:warm-robust:cold 11.580203 -11.3973423 34.557748 0.5492124

robust:warm-dwarf:warm 25.739167 1.5115849 49.966749 0.0330229

$`Morph:Ploidy`

diff lwr upr p adj

robust:4-dwarf:4 8.913188 -11.045812 28.872189 0.6440888

dwarf:6-dwarf:4 -19.458454 -43.799338 4.882430 0.1618438

robust:6-dwarf:4 57.235364 -17.127264 131.597993 0.1883769

dwarf:6-robust:4 -28.371642 -49.916773 -6.826511 **0.0049329**

robust:6-robust:4 48.322176 -25.172821 121.817172 0.3157319

robust:6-dwarf:6 76.693818 1.889864 151.497771 0.0423810

$`Treatment:Ploidy`

diff lwr upr p adj

warm:4-cold:4 7.994676 -10.96929 26.95864 0.6847028

cold:6-cold:4 -4.484656 -33.17066 24.20135 0.9762950

warm:6-cold:4 -6.380304 -33.76171 21.00110 0.9274601

cold:6-warm:4 -12.479333 -41.69173 16.73307 0.6756497

warm:6-warm:4 -14.374980 -42.30738 13.55742 0.5316629

warm:6-cold:6 -1.895647 -37.15857 33.36727 0.9989787

*Number of Stems per Plant*

*
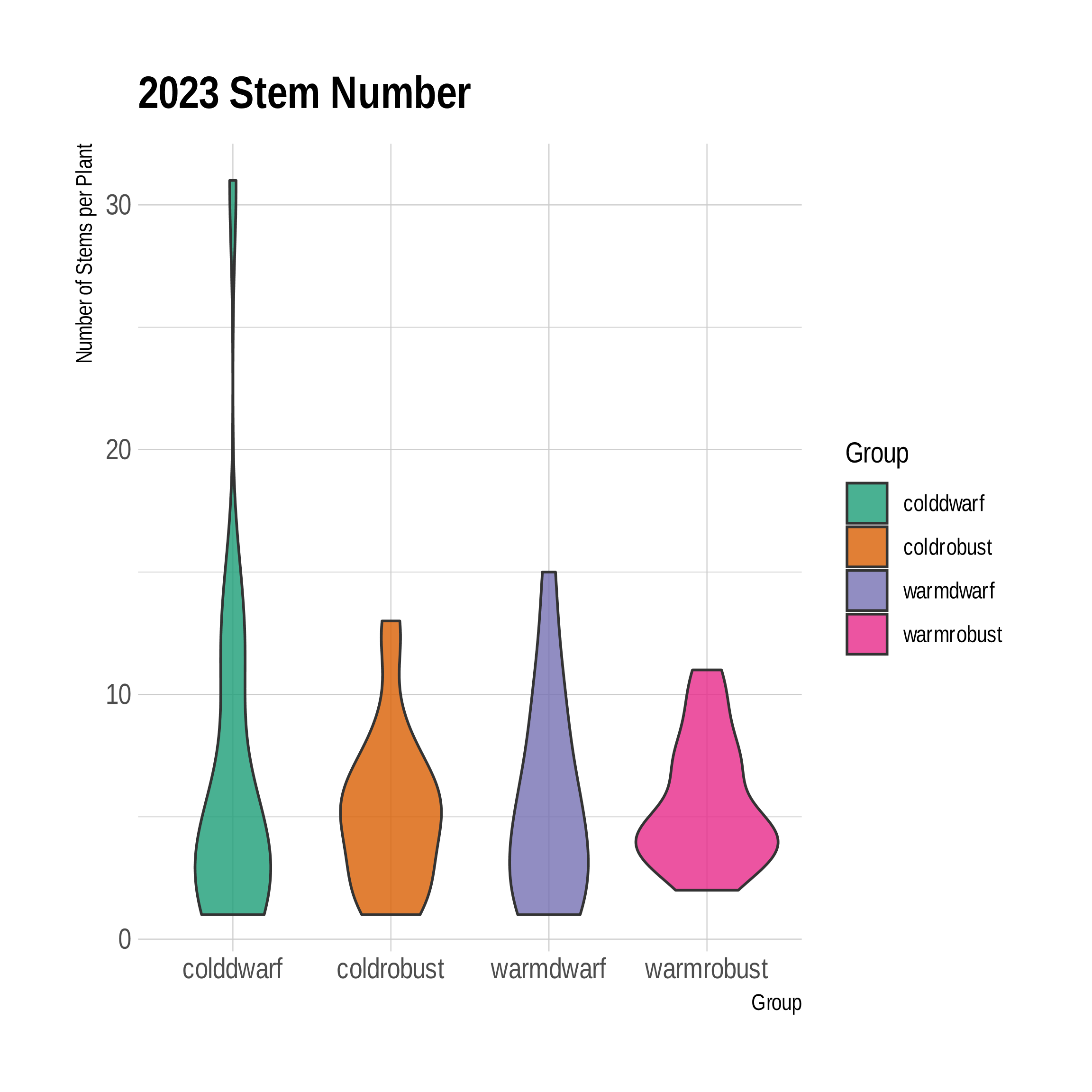
*

***Shapiro Wilk***

Shapiro-Wilk normality test

data: a_stems$n_St

W = 0.77732, p-value = **1.891e-09**

***Levene***

> leveneTest(a_stems$n_St, a_stems$Morph) #

Levene's Test for Homogeneity of Variance (center = median)

Df F value Pr(>F)

group 1 3.7994 0.05501 .

75

---

Signif. codes: 0 ‘***’ 0.001 ‘**’ 0.01 ‘*’ 0.05 ‘.’ 0.1 ‘ ’ 1

> leveneTest(a_stems$n_St, a_stems$Treatment) #

Levene's Test for Homogeneity of Variance (center = median)

Df F value Pr(>F)

group 1 0.8808 0.351

75

> leveneTest(a_stems$n_St, a_stems$Ploidy) #

Levene's Test for Homogeneity of Variance (center = median)

Df F value Pr(>F)

group 1 0.5077 0.4783

75

***AOV***

Df Sum Sq Mean Sq F value Pr(>F)

Morph 1 9.4 9.35 0.448 0.505

Treatment 1 2.4 2.38 0.114 0.736

Ploidy 1 54.8 54.78 2.626 0.110

Morph:Treatment 1 9.0 9.05 0.434 0.512

Morph:Ploidy 1 16.4 16.35 0.784 0.379

Treatment:Ploidy 1 4.8 4.80 0.230 0.633

Residuals 70 1460.2 20.86

***Tukey HSD***

Tukey multiple comparisons of means

95% family-wise confidence level

Fit: aov(formula = n_St ~ (Morph + Treatment + Ploidy)^2, data = a_stems)

$Morph

diff lwr upr p adj

robust-dwarf -0.7 -2.784777 1.384777 0.5052732

$Treatment

diff lwr upr p adj

warm-cold -0.3525068 -2.433042 1.728028 0.7364355

$Ploidy

diff lwr upr p adj

6-4 -1.796322 -4.354869 0.7622248 0.16585

$`Morph:Treatment`

diff lwr upr p adj

robust:cold-dwarf:cold -1.36081847 -5.143529 2.421892 0.7797377

dwarf:warm-dwarf:cold -1.10252348 -5.167749 2.962702 0.8913102

robust:warm-dwarf:cold -1.08382391 -5.037483 2.869835 0.8882173

dwarf:warm-robust:cold 0.25829499 -3.586321 4.102911 0.9980177

robust:warm-robust:cold 0.27699456 -3.449456 4.003445 0.9973225

robust:warm-dwarf:warm 0.01869957 -3.994228 4.031628 0.9999993

$`Morph:Ploidy`

diff lwr upr p adj

robust:4-dwarf:4 -1.9479575 -5.226414 1.330499 0.4058098

dwarf:6-dwarf:4 -2.8281231 -6.933800 1.277554 0.2760032

robust:6-dwarf:4 -0.4399604 -12.756991 11.877070 0.9996995

dwarf:6-robust:4 -0.8801657 -4.507335 2.747004 0.9191333

robust:6-robust:4 1.5079970 -10.657899 13.673893 0.9879085

robust:6-dwarf:6 2.3881627 -10.026238 14.802563 0.9573313

$`Treatment:Ploidy`

diff lwr upr p adj

warm:4-cold:4 -0.3971463 -3.495671 2.701378 0.9866692

cold:6-cold:4 -2.2933043 -7.282320 2.695711 0.6227754

warm:6-cold:4 -1.7847473 -6.290682 2.721187 0.7251924

cold:6-warm:4 -1.8961580 -6.994394 3.202078 0.7619060

warm:6-warm:4 -1.3876010 -6.014175 3.238973 0.8590560

warm:6-cold:6 0.5085570 -5.549050 6.566164 0.9961582
